# Needs among persons with advanced COPD and their informal caregivers: a multi-perspective qualitative study focusing on the potential role of palliative care

**DOI:** 10.1186/s12890-026-04828-5

**Published:** 2026-10-19

**Authors:** Kira Schmidt-Stiedenroth, Julia Salandi, Alexandra Schmidt, Balam Er Dedekarginoglu, Stefanie Paniczek, Manuela Schallenburger, Anette Scholz, Jacqueline Schwartz, Kathrin Fricke, Stefanie Keymel, Achim Mortsiefer, Martin Neukirchen, Adrian Loerbroks

**Affiliations:** 1 Institute for Occupational, Social, and Environmental Medicine, Centre for Health and Society, Medical Faculty and University Hospital Düsseldorf, Heinrich Heine University Düsseldorf, Düsseldorf, Germany; 2 Institute of General Practice and Primary Care (iamag), Chair of General Practice II and Patient-Centredness in Primary Care, Witten/Herdecke University, Witten, Germany; 3 Clinic for Cardiology, Pulmonology & Angiology, Medical Faculty and University Hospital Düsseldorf, Heinrich Heine University Düsseldorf, Düsseldorf, Germany; 4 Interdisciplinary Centre of Palliative Care, Medical Faculty and University Hospital Düsseldorf, Heinrich Heine University Düsseldorf, Düsseldorf, Germany; 5 Institute for eHealth and Healthcare Management, University of applied science, Flensburg, Germany; 6 Clinic of Pneumology and Allergology, Center for Sleep Medicine and Respiratory Care, Institute of Pneumology, Bethanien Hospital, University of Cologne, Solingen, Germany; 7 Department of Anaesthesiology, Medical Faculty and University Hospital Düsseldorf, Heinrich Heine University Düsseldorf, Düsseldorf, Germany

**Keywords:** COPD, Needs, Palliative care, Health services research, Patient-oriented healthcare, Qualitative research, Germany

## Abstract

**Objectives:**

To explore needs among persons with advanced COPD and informal caregivers, and to explore how palliative care can address those needs.

**Background:**

COPD is a common, progressive disease affecting lung function and represents a leading cause of death. Advanced COPD is associated with a high symptom burden and poor quality of life. Persons with advanced COPD may benefit from palliative care, but its use among persons with COPD remains limited, also in Germany.

**Methods:**

A qualitative, multi-perspective interview study was conducted with persons suffering from advanced COPD, informal caregivers, and formal caregivers (general practitioners, pulmonologists, and palliative care physicians). A total of 53 interviews were analysed following structuring (Kuckartz’) as well as latent content analysis methods using MAXQDA.

**Results:**

Key needs were identified in the following domains (1) management of dyspnoea and anxiety, (2) home-based healthcare, (3) emergency planning and support, (4) psychosocial care, (5) organisational support, and (6) timely information about COPD and palliative care. Persons who had previously received PC reported various needs met in the first five areas.

**Conclusions:**

Persons with advanced COPD and their informal caregivers reported numerous needs. Persons receiving palliative care reported relief from various needs. Timely information about COPD’s progression and palliative care could increase acceptance and thus use of palliative care among these persons and could consequently reduce or curtail the burden of living with advanced COPD.

**Supplementary Information:**

The online version contains supplementary material available at https://doi.org/10.1186/s12890-026-04828-5.

## Background

 Chronic obstructive pulmonary disease (COPD) is a common disease with a worldwide prevalence estimated at 10.6%, the equivalent of 480 million cases [1]. It constitutes a leading cause of disability and is the third cause of death around the globe [1, 2]. COPD is characterized by a chronic and progressive disruption of the airway function of the lungs by bronchial obstruction that is not fully reversed by bronchodilators [3]. The Global Initiative for Chronic Obstructive Pulmonary Disease (GOLD) classifies the severity of COPD according to the severity of airflow obstruction, the number of exacerbations in the previous year, as well as the symptom burden [4]. Following the GOLD classification, advanced COPD can be defined by a very severe airflow obstruction (FEV_1_ < 30% predicted, corresponding to GOLD 4) and/or a high symptom burden, including one or more moderate or severe exacerbations during the previous year (corresponding to GOLD E) [4]. Persons with advanced COPD frequently report progressive dyspnoea and related fear and anxiety as well as pain, fatigue, and poor symptom control [5, 6], functional impairment, and a poor quality of life [7, 8]. The mobility restrictions caused by these symptoms further impair the patients’ autonomy in everyday life, often contributing to feelings such as isolation and loneliness [5, 9–11]. This burden also affects the informal caregivers of persons with advanced COPD. Informal caregivers report a negative impact on their quality of life, e.g., due to the often isolating and stressful caregiver role [12–14]. However, informal caregivers are often overlooked by healthcare providers in the context of COPD [14].

Several studies have explored the needs of persons with COPD. These include needs related to understanding their disease, managing symptoms and medication, organisational and financial support, managing relationships and social life, and access to professional care [15, 16]. As for persons with advanced COPD, the need to manage dyspnoea has been considered crucial [17]. More recent studies have also explored the needs of their informal caregivers, emphasizing mental support, caregiving skills, supportive social structures, and reducing the caregiving burden [18]. It has been shown that palliative care (PC) can help fulfil these needs [19, 20]. PC is defined by the World Health Organization as “an approach that improves the quality of life of patients and their families who are facing problems associated with life-threatening illness” [21]. PC is multidisciplinary, multiprofessional, holistic, and person centred [22, 23], and is supposed to involve informal caregivers by definition. PC in patients with advanced COPD can be carried out in parallel with disease-specific pulmonological treatment. The European Respiratory Society (ERS) recommends considering an additional PC approach when patients with COPD and their relatives have unmet physical, psychological, social, or spiritual/existential needs [22]. According to the ERS, these needs should be assessed based on reports from the patients and their relatives and/or screening questionnaires [22]. Also, disease severity and/or healthcare utilization (e.g., hospital admissions or advanced respiratory therapies) may provide useful guidance [22].

In Germany, PC is provided as general outpatient palliative care (GOPC), specialized outpatient palliative care (SOPC), or inpatient palliative care. Inpatient PC can also be general or specialized. While GOPC can be carried out by GPs, specialists, and care services, SOPC is provided by specialized PC-teams and is indicated in patients with complex symptoms where GOPC is not sufficient. Persons with COPD usually receive outpatient healthcare from office-based physicians (i.e., general practitioners and pulmonologists). These physicians may initiate both GOCP or SOPC, depending on the symptom burden.

Studies from other countries report that PC remains underutilized among persons with COPD [11, 24–26]. While PC is well established for oncological diseases, access for persons with advanced COPD remains limited due to various barriers. Among those are misunderstandings about PC, such as the belief that it is only available for persons with cancer, and uncertainties related to the adequate time to initiate PC for persons with COPD [11, 22, 24–26]. In Germany, PC needs among persons with COPD and the role of PC in COPD remain understudied [27], even though the National Care Guideline for COPD explicitly promoted the understanding of COPD “as a chronic disease that requires ongoing care, including palliative care” [28].

Thus, the present paper aims to fill this gap by exploring the PC needs among persons with advanced COPD and their informal caregivers in the context of the German healthcare system. We addressed the following research questions: (1) What are the (PC) needs of persons with advanced COPD and their informal care givers? and (2) How are these needs addressed by PC in the context of the German healthcare system? The expected insights will likely help to better understand the potential role of PC in the healthcare delivery of persons with advanced COPD and their informal caregivers, and can inform the formulation of adequate practice recommendations aiming at improving the healthcare provision of these persons.

## Methods

### Study design

We conducted a multi-perspective, qualitative interview study. Qualitative studies aim to explore the range of perspectives of a given topic, and sample sizes are typically smaller than quantitative studies [29]. In addition to the views of persons with advanced COPD and their informal caregivers, we also aimed to capture the perspectives of formal caregivers. General practitioners (GPs) and pulmonologists are mainly responsible for the healthcare delivery of persons with advanced COPD and for the initiation of PC. PC physicians are experienced regarding the needs of persons with life-threatening illnesses.

Exploring PC needs can be difficult if the potential beneficiaries or providers are not fully aware of what PC actually is or what it can offer [30]. For this reason, a qualitative approach sensitive to needs implicit in the burden experienced by persons with advanced COPD and their informal caregivers can be particularly useful or even necessary. Expanding beyond felt and expressed needs [31] and further considering normative needs (i.e., the perspective of formal caregivers) [31], as well as gaps in the experiences of persons already receiving PC compared to those who have not, can help identify needs not recognized as such until they are fulfilled, namely, comparative needs [31]. Thus, while we aimed to capture healthcare needs in general, we also focused on how PC fulfils, or may help fulfil, the identified needs. Thus [29], we included persons with advanced COPD and informal caregivers who have not received PC care (yet), and persons who already received PC.

Ethical approval was provided by the Ethics Committee at the Medical Faculty of Heinrich Heine University Düsseldorf (Identifier: 2023–2399). Written informed consent was obtained from all participants prior to participation in this study. Reporting was done following the COREQ framework [32].

### Participant selection and recruitment process

A chain referral recruitment system was applied in this study. First, we contacted formal caregivers (i.e., GPs, pulmonologists, and PC physicians) either directly, e.g. via e-mail, or indirectly via advertisements of our study in social media, mailing lists, and the newsletters of different professional associations and networks. These included the General Practitioner Research Practice Network (HAFO Netzwerk) and all GP practices associated with the IAMAG Institute in Witten/Herdecke through teaching or research, the Network of General Practitioners in Duesseldorf (HAND e.V.), and the German Society of Palliative Care. Additionally, physicians who had participated in previous studies of the collaborating institutions were contacted, and members of the research team drew on their professional networks to disseminate information about the study.

The inclusion criteria for physicians included: (1) a completed or almost finished specialization in family practice, pulmonology, and/or palliative care and (2) sufficient knowledge of German to participate in an interview. Physicians were invited to participate in a semi-structured interview and/or to recruit patients. They received a monetary incentive for each successfully recruited patient, and also for their own participation in an interview. Because the number of physicians reached remains unknown, a response rate cannot be calculated.

The recruitment of persons with advanced COPD was done through their formal caregivers in order to make sure that patients met the selection criteria, namely: (1) a diagnosis of advanced COPD, which was defined as: FEV_1_ (forced expiratory volume in one second) < 30%, GOLD (Global Initiative for Chronic Obstructive Lung Disease, Updated 2023) group E and/or stadium 4, plus answer “no” to the surprise-question (“Would you be surprised if this patient were to die in the next twelve months?”) by the recruiting physicians, (2) legal age, and (3) sufficient knowledge of German to participate in an interview. Exclusion criteria for patients were: (1) the presence of one or more comorbidities for which palliative care was already being received, or the presence of one or more comorbidities which could be lethal within the next twelve months (e.g., oncological diseases), (2) the physical or cognitive inability to participate in an interview. Persons meeting the inclusion criteria received detailed information about the study, a written informed consent form, and two recruiting packages for informal caregivers from the recruiting physicians. They could participate regardless of whether they had an informal caregiver or not. Informal caregivers were recruited by the persons with COPD. They did not need to be relatives of the patients but had to be involved in caring activities. In this way, we ensured that only informal caregivers of patients meeting our inclusion criteria would participate. Inclusion criteria for informal caregivers further were (1) legal adult, and (2) sufficient knowledge of German to participate in an interview. Shopping vouchers were raffled among persons with advanced COPD and the informal caregivers who participated in the interviews. Recruitment and data collection took place between February and November 2024.

### Data collection

Taking into consideration needs presented in previous studies on persons with (advanced) COPD [5, 6, 15, 16, 33–36] and our goal to capture multiple perspectives, we developed four interview guides addressing the following groups: (1) Persons with advanced COPD, (2) informal caregivers of persons with advanced COPD, (3) GPs and pulmonologists, and (4) PC physicians. GPs and pulmonologists tend to work in similar settings in Germany, that is, as office-based physicians, while palliative care physicians do not tend to work in such a setting, thus warranting separate interview guides (see supplementary tables 1 to 4). Preliminary interview guides were developed by KS and jointly discussed with team members who are either experts in Public Health and Health Services Research (AL, ASchm, JSa), Public Health and Nursing Science (MS), or are themselves specialist physicians (AScho and AM are GPs, BED and SK are pulmonologists, and JSch, MN and SP are PC physicians).

All interviews were conducted by KS in German. KS holds a PhD in Medical Anthropology and has extensive experience in qualitative research, including semi-structured interviews and research in healthcare settings [37, 38]. At the time of conduction of this study, KS was employed as research associate and pursuing a Master’s degree in Public Health. KS conducted a pilot test interview with a GP, after which the GP provided feedback. Since the interview went well, it was also included in the analysis. The interview guides were slightly adapted during the course of data collection. Most interviews were conducted over the phone, with the exception of three interviews with persons with advanced COPD conducted face-to-face; one in a PC unit in a hospital and two in the interviewees’ homes. Interviews were conducted only once and individually, only in one case a relative was present during an interview with a patient. There was no relationship between KS and the interview participants previous to recruitment for this study.

All interviews were digitally audio recorded and consequently transcribed. After each interview, persons with advanced COPD and informal caregivers were asked a set standardized questions to collect background information on participants’ gender, education, ethnical background, and symptom burden (see Supplementray Table 1). Physicians were asked to subjectively assess their professional experience with patients with COPD and the years of experience as physicians.

### Data analysis

The analysis of the interview transcriptions was conducted following Kuckartz’ structuring content analysis approach [39] using the Software MAXQDA 2024. During the interviews and also during analysis it became apparent that some persons with advanced COPD and informal caregivers did not explicitly recognize needs when directly asked, even though such needs seemed apparent from a contextualization of their statements. Rather than articulating explicit needs, participants often framed them as challenges, limitations, or issues related to living with COPD. Thus, we further complemented structuring content analysis with latent content analysis [40] in order to also identify and include implicit and comparative needs [31]. Latent content analysis asks what intended to be said with the goal of finding the underlying meaning of a text, thus it differs from manifest analysis in that it requires interpretation by the coders [40]. Using latent content analysis, we also operationalized needs via interpretation, for example when participants described challenges or limitations that could potentially be addressed through PC, or when they described how PC had fulfilled a need. The interviews were coded by three female team members (KS, JSa and ASchm) with training in Medical Anthropology, Psychology and Social Science, respectively, and extensive experience with qualitative research in the medical field. Three female specialist physicians (AScho, BED, and SP) assisted during the analysis of the interviews by clarifying questions and helping refine the code system.

In the first coding round, KS and JSa coded each the same five interviews building main categories deductively according to the interview guides and adding inductive sub-categories emanating from the material. The coding system was discussed and adjustments regarding the overlapping and clarity of sub-codes were made. KS coded the same five interviews and further eight ones with the adjusted coding system in a second coding round and consequently discussed its application with JSa. There was agreement that sorting the main categories by the target groups of the interview guides lead to the unnecessary repetition of many sub-codes, hindering a clear analysis. Thus, it was decided to establish new main categories based on the leading research questions instead of the interview guides, namely: (1) Needs of patients and informal caregivers, (2) Normative needs from formal caregivers’ perspective, and (3) barriers to access PC. KS then coded the same 13 interviews in a third round with the reworked coding system, sorting existing sub-codes within one of the three main categories and new sub-codes were further introduced inductively. Subsequently, ASchm coded eight interviews with GPs and KS coded further 32 interviews with persons with COPD, their informal caregivers, pulmonologists and palliative care physicians. Data saturation, defined as the point where additional data no longer provided novel information relevant to answer our research questions [41], was reached after coding 53 interviews. Once coding was finished, the material was again reviewed and needs were structured by KS in a needs catalogue. The needs catalogue was revised by the research team, resulting in the domain categories presented in the results section. Due to the richness of the data gathered and space constraints, findings regarding access barriers to PC are reported in a separate paper. The transcripts and findings were not returned to participants for comments or feedback.

The verbatim quotes presented in the findings section and in the supplementary materials were translated from the original German with the support of artificial intelligence tools (ChatGPT and DeepL) and were verified by KS, who proofed their accuracy.

## Findings

We included 53 interviews in the qualitative content analysis. To reasonably limit the scope of this paper, only topics relevant to the needs of persons with advanced COPD and their informal caregivers are reported here. We also included normative needs from the perspective of formal caregivers, and the role of PC in fulfilling those needs. Not all needs were explicitly mentioned in the interviews. Thus, to better capture and contextualize them, the burden experienced by persons with advanced COPD and their informal caregivers, as well as the relief of those burdens experienced by persons who received PC, were also included in the results. This is an important aspect of latent content analysis, which is more interpretative than merely manifest analyses [40].

### Demographics

#### Persons with advanced COPD

We included 17 persons diagnosed with advanced COPD. The mean duration of their interviews was 31 minutes (range: 13:59–51:08). The average age among participants of this group was 68 years, with women and men almost equally represented (see Table 1). Persons with advanced COPD reported an average EuroQol five dimensions of health-Visual Analogue Scale (EQ-5D-VAS) score of 25.8 (range 0-100). All persons with COPD—except for one missing for whom a Clinical COPD Questionnaire (CCQ) score could not be calculated—reported a CCQ score above 1.4 (CCQ score range: 0–6). This value is considered a cut-off indicating general poor health and substantial limitation or symptoms, as well as severe impairment in daily life due to COPD [42]. Regarding mental health, more than half persons who answered the Generalized Anxiety Disorder-2 (GAD-2) questionnaire may have a generalized anxiety disorder according to their score [43]. As for depression symptoms, six out of 15 persons who answered both questions of the Patient Health Questionnaire-2 (PHQ-2) presented a score indicating major depression symptoms [44]. Thirteen persons with advanced COPD agreed or fully agreed with the statement “Do you feel adequately supported overall with regard to your COPD?”. Among this group, five persons had or were receiving PC at the time of the interview, while the remaining 13 declared not to have received PC yet. All patients in our study were living in their homes.

#### Informal caregivers

A total of ten informal caregivers were included in the analysis (see Table 1). They were commonly the spouse or partner of a person with advanced COPD, their children or, in one case, an acquaintance/friend. Some of the children were supported by their own spouses in taking care of the person with advanced COPD. Three informal caregivers declared to be affected by a serious disease themselves, and one reported to be also responsible for the care of one child. The interviews of this group lasted on average 40 minutes (range: 19:42–01:19:42). Most informal caregivers were women, middle-aged, married, had a high educational level, and lived in a city. Almost half of them were first- or second-generation immigrants. Regarding their mental health, four informal caregivers reported anxiety symptoms (GAD-2 score ≤ 3), while three reported depression symptoms (PHQ-2 score ≥ 2). Half of the informal caregivers agreed or fully agreed with the statement: “Do you feel adequately supported in your role as a supporter/caregiver of a person with advanced COPD?”. Six out of ten informal caregivers included in our analyses were, at the time of the interview, caring for a person receiving PC.

**Table 1:** Characteristics of persons with advanced COPD and informal caregivers

Variables	Persons with advanced COPD (*n* = 17)	Informal caregivers (*n* = 10)
Female gender, n (%)	8 (47.05)	7 (70.00)
Age, mean (SD)	68.65 (7.23)	53 (14.09)
Familial situation, n (%)		
Married or living in a partnership	13 (76.47)	8 (80.00)
Divorced	4 (23.52)	1 (10.00)
Single or widowed	0 (0.00)	1 (10.00)
School education, n (%)		
Low (*Hauptschule*)	8 (47.05)	3 (30.00)
Middle (*Realschule Polytechnische Hochschule*)	5 (29.41)	2 (20.00)
High (*Fachhochschulreife, Abitur*)	4 (23.52)	4 (40.00)
Prefer not to say	0 (0.00)	1 (10.00)
Place of living^†^, n (%)		
Small town	8 (47.05)	4 (40.00)
City	9 (52.94)	6 (60.00)
Migration background^*^, n (%)	3 (17.64)	4 (40.00)
Employment, n (%)		
Full-time	1 (5.88)	4 (40.00)
Part-time	0 (0.00)	1 (10.00)
Retired	16 (94.11)	5 (50.00)
Care level, n (%) None 1 2 3 4 5	2 (11.76) 0 (0.00) 5 (29.41) 5 (29.41) 4 (23.52) 1 (11.76)	Not applicable
Person with COPD lives permanently in the own household, n (%)	Not applicable	5 (50.00)
Report to have themselves a severe disease	Not applicable	3 (30.00)
EQ-5D-VAS score, mean (SD)	25.8 (21.81)	Not applicable
CCQ score ≥ 1.4, n (% of valid answers)	16 (100)	Not applicable
GAD-2 score ≥ 3 (n, % of valid answers)	9 (56.25)	4 (40.00)
PHQ-2 score ≥ 2, n (% of valid answers)	6 (40.00)	3 (30.00)
COPD support^‡^, n (%) Fully disagree Disagree Neither agree nor disagree Agree Fully agree	1 (5.88) 0 (0.00) 1 (5.88) 3 (17.64) 11 (64.70)	1 (10.00) 2 (20.00) 2 (20.00) 3 (30.00) 2 (20.00)

Abbreviations and acronyms: COPD: Chronic obstructive pulmonary disease; n: Number; SD: Standard deviaton; EQ-5D-VAS: EuroQol 5 Dimensions Visual Analogue Scale; CCQ: Clinical COPD questionnaire; GAD-2: Generalized Anxiety Disorder-2; Patient Health Questionnaire-2

† Small town: 20.000-100.000 inhabitants; city: >100.000 inhabitants

*Person herself and/or or at least one parent was born outside of today’s German boundaries

**‡ Question for patients:** “Do you feel adequately supported overall with regard to your COPD?”; question for informal caregivers: “Do you feel sufficiently supported in your role as a caregiver of a person with advanced COPD?”, 5-point Likert scale (1=fully disagree to 5=fully agree)

#### Formal caregivers

In total, we conducted individual interviews with 26 formal caregivers: ten GPs, nine pulmonologists and seven PC physicians. On average, formal caregivers were middle aged (see Table 2). Most of them were women with around 20 years of experience as a physician in average. Only one GP and one pulmonologist had completed additional training in PC. The average self-rated experience with COPD patients was highest among pulmonologists, while the lowest was among PC physicians. GPs presented the highest agreement with the statement “Patients with end-stage COPD receive adequate palliative care”, while no PC physician agreed with the statement.

**Table 2:** Characteristics of formal caregivers

Variables	General practitioners (*n* = 10)	Pulmonologists (*n* = 9)	Palliative care physicians (*n* = 7)
Female, n (%)	7 (70.00)	6 (66.66)	5 (71.42)
Age, mean (SD)	51.1 (11.61)	45.88 (8.46)	50.87 (8.35)
Are additionally palliative care physicians, n (%)	1 (10.00)	1 (11.11)	NA
Self-rated experience with COPD^†^ Advanced and very experienced (4–5), n (%)	6 (60.00)	9 (100)	4 (57.14)
Years of experience as physician, mean (SD)	22.4 (9.22)	18.44 (8.53)	21.14 (6.86)
Years of experience as PC physician, mean (SD)	NA	NA	8.28 (5.40)
Palliative support for end-stage COPD^‡^ Agree and fully agree (4–5), n (% of valid answers)	4 (44.44)	1 (11.11)	0 (0.00)

**Abbreviations and acronyms**: n: Number; SD: Standard deviaton; COPD: Chronic obstructive pulmonary disease; PC: palliative care

^†^ Question “How do you assess your own experience with COPD?”, 5-point Likert scale (1 = no experience at all to 5 = very experienced)

^‡^ Question: “To what extent do you agree with this statement? ‘Patients with end-stage COPD receive adequate palliative care.’”, 5-point Likert scale (1 = fully disagree to 5 = fully agree)

### Needs of persons with advanced COPD and their informal caregivers

Most of the needs among persons with advanced COPD and their informal caregivers as well as the normative needs mentioned by formal caregivers can be categorized into six domains of healthcare provision: (1) management of dyspnoea and anxiety, (2) home-based healthcare, (3) emergency planning and support, (4) psychosocial care, (5) organizational support, and (6) timely information about COPD and PC. Psychosocial care and organizational support were deductive categories we explicitly probed during the interviews, whereas the remaining categories were constructed inductively. In the following, we present the main findings with a selection of exemplary quotes. Table 3 presents an overview of the burdens and (normative) needs of persons with advanced COPD and their informal caregivers, structured by domains and their corresponding codes. It is important to emphasize that needs are interconnected and should therefore not be understood as clearly distinct categories, but rather as overarching needs. Relevant codes from the coding scheme with representative verbatim quotes not presented in the text can be found in the supplementary tables 1 to 4. The quotes highlight several topics, underscoring the interconnectedness of needs.

**Table 3:** Overview of findings: relevant needs categorized by domains and their corresponding codes.*

Domains of healthcare provision	Burden and needs of persons with advanced COPD, codes	Burden and needs of informal caregivers, codes	Perspective
Management of dyspnoea and anxiety	• Anxiety/fear of suffocation/panic • Being a burden for others • Breathing techniques / non-pharmacological therapies • Denial/avoidance • Dependence on others • Dyspnoea • Loss, longing or sadness • Limited mobility • Limited social interactions • Medications less helpful than before • Needing assistance for daily activities	• Responsibility for patient’s wellbeing	Persons with advanced COPD and their informal caregivers
	• Breathing techniques/non-pharmacological therapies • Inhalation technique • Palliative care • Pharmacological therapies		Formal caregivers
Home-based healthcare	• Anxiety/fear of suffocation/panic • Dyspnoea • Limited mobility • Needing assistance with daily activities • Home-based healthcare		Persons with advanced COPD and their informal caregivers
	• Home-based healthcare		Formal caregivers
Emergency planning and support	• Breathing techniques / non-pharmacological therapies • Emergency kit • Instructions to handle emergencies/exacerbations	• Instructions to handle emergencies/exacerbations	Persons with advanced COPD and their informal caregivers
	• Emergency care in case of exacerbations • Professional contact in case of emergency • Palliative care		Formal caregivers
Psychosocial care	• Anxiety/fear of suffocation/panic • Being a burden for others • Denial/avoidance • Dependence on others • Limited social interactions • Loss, longing or sadness • Needing someone to talk to	• Acceptance • Anger • Emotional support for patient • Frustration • Limited social interactions • Loss, longing or sadness • Patient refuses psychosocial support • Preparing for end-of-life • Responsibility for patient’s wellbeing • Putting patient’s needs first • Time consuming care activities	Persons with advanced COPD and their informal caregivers
	• Psychological/psychiatric support • Palliative care • Support groups	• Psychological/psychiatric support • Pharmacological therapies • Support with care duties • Support groups	Formal caregivers
Organizational support	• Limited mobility • Needing assistance with daily activities	• Burdened by severity of COPD • Dealing with bureaucracy • Nursing care support is not sufficient • Organizational support is not sufficient • Time consuming care activities • Responsibility for chores	Persons with advanced COPD and their informal caregivers
	• Case management • Coverage of medical devices • Family involvement • Nutritional advice • Organizational support • Lifestyle changes	• Housekeeping support • Support with care duties	Formal caregivers
Timely information about COPD and palliative care	Information about COPD Information about risk exposure	• Information about COPD • Preparation later stage • Surprised about severity	Persons with advanced COPD and their informal caregivers
	• Challenging communication • Early information • Information about COPD • Information about palliative care	• Information about COPD • Information about palliative care	Formal caregivers

* Because domains are interrelated, some codes overlap

#### Management of dyspnoea and anxiety

Persons with advanced COPD and their informal caregivers described their daily lives as being shaped by a variety of symptoms, among which dyspnoea was generally considered the most burdensome one, both physically and psychologically:

*Oh, it’s terrible, no one can really imagine it. You truly feel like you’re suffocating every single moment. Even when someone talks to you calmly, it’s just AWFUL, I can’t even describe it. You feel like any moment you’re going to be gone. […]* [Patient, female].

Dyspnoea was mentioned to cause considerable delays and difficulties in performing everyday activities such as getting dressed or washing oneself, to affect mood, and to cause fear of suffocation, anxiety, and panic attacks. Dyspnoea also was considered a burden by informal caregivers who had to assist persons with advanced COPD with brooming and other daily tasks (ST2: Q1, Q2). These symptoms severely restricted the mobility and social life of persons with advanced COPD and their informal caregivers, as the lives of patients increasingly took place within their own home (ST1: Q1, Q2). Negative feelings such as being a burden to others, being dependent on others, feelings of loss, longing, and sadness for not being able to take part in (social) life anymore, and worrying about future exacerbations were commonly mentioned by persons with advanced COPD (ST1: Q3, Q4, Q5):

The feeling is that when someone is limited, they can no longer take part in things at all. I used to be right in the middle of public life. Yes, I worked in the hotel industry and dealt with many celebrities, and I had seen the world, travelled and, and, and. And suddenly, it’s all gone. Suddenly, everything is gone because of this illness. Yes, and that is / you ask yourself, why is it all like this? Without my wife and my daughter, I probably / I don’t know. I’ve withdrawn. Not because I wanted to, but because I simply can’t take part in life anymore the way I would like to. You know? [Patient, male].

Persons with advanced COPD also reported that medications that worked well to manage dyspnoea and anxiety during previous stages of the disease no longer helped as expected, delaying relief and further limiting them (ST1: Q6). Formal caregivers explained that PC could offer relief of dyspnoea and anxiety through opioids and anxiolytics, as well as devices that aid inhalation through nebulization, soothing conversations, and psychosocial care (ST3: Q1, Q2, Q3). However, it was reported that some physicians thought that morphine was contraindicated for dyspnoea, and that reservations regarding the addictive behaviour of persons with advanced COPD prevented some GPs and pneumologists from prescribing opioids. Persons with advanced COPD and informal caregivers expressed relief using pharmacological and non-pharmacological therapies such as pursed-lip breathing provided in the context of PC (ST4: Q1, Q2, Q3), and emphasized the positive social aspects of lung sports groups. Both formal and informal caregivers pointed out that appropriate instructions and reminders of how to use inhalers and breathing techniques are needed, and that such training should ideally take place at home (ST1: Q8, ST3: Q4, Q5). Non-pharmacological therapies such as those taught in lung sports groups seemed especially important in view of temporary shortages of medicines treating dyspnoea, which were reported by both patients and physicians as a major problem. However, this kind of therapy was not always available to patients, for example because there were no service providers close to the patients, because the physicians admittedly not always thought of prescribing it, because it was hard to find therapists doing home visits or lung sports groups in rural areas, or because training was not covered by the insurance (ST3: Q6, Q7, Q8). Psychological support was also considered necessary for the management of anxiety and fear of suffocation; this need is discussed in the section psychosocial care.

#### Home-based healthcare

Advanced COPD limited mobility to the extent that some persons reported to hardly leave their homes anymore or to have not done so in years (ST1: Q1, Q2). Attending physicians’ appointments was described as very stressful and in some cases being only possible with special arrangements such as a non-emergency medical patient transport (ST1: Q9). Some persons with advanced COPD relied on home visits by their GPs and, in case of exacerbations or other acute worsening of the health condition, to rely on emergency doctors or calling an emergency ambulance to receive healthcare at the hospital. Going to the doctor or relying on the ambulance was considered very distressing, also for informal caregivers:

What’s important is that there’s also medical care on site. Because I can’t do it anymore, I just can’t. […] In the waiting room, we sat there for four hours, I collapsed, and then they finally took me in. And now, last time we were there again, it was hell again. […] I don’t want that anymore. It’s so important, I can’t always call an ambulance. What I need is for help to be given to me at home. My wife can’t do everything either, we can’t. She says, “I’ll do it, I’ll do it,” but I can see it in her, it’s getting harder and harder. Because she suffers from it too. Because she doesn’t really know what to do anymore [Patient, male, ].

Formal caregivers underscored the need for home visits from the healthcare provider perspective, but clarified that not all GPs conduct home visits, and that pulmonologists do not do home visits at all (ST3: Q9, Q10). Consequently, many persons with advanced COPD were no longer regularly seen by pulmonologists, except when they went to the hospital for emergencies. Formal caregivers expressed that some needs, such as the need for psychological support, may be overlooked in emergency settings. Formal caregivers further remarked that in the current German healthcare system, there are no sufficient psychotherapists or physiotherapists doing home visits, adding to the lack of basic services covering needs of patients with restricted mobility due to COPD (ST3: Q11). To fill the gap of home-based healthcare needs, it was suggested that non-physician medical staff such as medical assistants could be sent to patients with advanced COPD (ST3: Q4), or that telemedicine should be used. Specialized PC teams visiting persons with advanced COPD at home and the emergency medications provided by them fulfilled the need for home-based healthcare and for management of dyspnoea and anxiety, providing reassurance and confidence:

So, for me, it [palliative care] is more reassuring. And it also makes me feel more confident, because now I can help my husband at home. And if it really doesn’t work anymore, then they come by as well, right? And that works, right? Before, we only had inhalers and sprays, and those just weren’t enough. Now he’s getting some medications that / to help him breathe better and also / to help him relax. And I see that as a positive. Because of that, my husband does better here at home and doesn’t have to go to the hospital. […] [Informal caregiver, female].

#### Emergency planning and support

All persons with advanced COPD were familiar with exacerbations. Some reported to worry about exacerbations happening again (ST1: Q10). Emergencies due to exacerbations were described as very distressing for informal caregivers. They often hesitated before calling emergency medical services, especially when the patients were objecting (ST2: Q3). Persons with advanced COPD expressed that, in hindsight, they had waited too long before calling emergency services during a previous exacerbation (ST1: Q10, Q11).

The role of informal caregivers in case of exacerbations was described from positive to difficult, depending how they were able to handle the situation. Some informal caregivers expressed not to know exactly what to do the in case of exacerbations to best support patients and wished for such information to be given to them by healthcare providers (ST2: Q4). Formal caregivers mentioned the need for patients and informal caregivers to have someone to talk to in case of an emergency, as some supporting and soothing words may go a long way to relief dyspnoea, anxiety and panic in such situations, as alternative to or in addition to medication, for example while waiting for them to have an effect (ST3: Q1):

I think, on the one hand, it’s really about the / I mean, being able to speak to someone in case something happens, to take away the worry about that, that someone is available, I mean permanent accessibility. And then, of course, also the medication therapy: anti-anxiety medication and medication to treat shortness of breath [GP, female, 8 years of experience].

Persons who had received some sort of emergency medications or the 24/7 emergency contact number of a PC team reported positively about knowing that they had fast acting drugs and someone to talk to (ST1: Q12):

So, medication, that has been my life insurance, and it still is today. But now I also know that I can count on human help. Namely, the palliative care team. They also said: “Mr. [name], if anything happens, you / There’s an emergency number.” And I really should call if things are really hitting the fan for me. I’ve never made use of it yet, but if it really comes to that, then I will. Because I know that I’ll get help. And even if it’s just some calming words at first, or they’ll also come here. I also have the morphine that you can basically have injected into your belly, they set all of that up for me as well. I don’t do it myself. If I need it, then I’m supposed to call, and someone will come and administer it. But everything is right here within reach. And I’m set up here like in a hospital. […] Exactly, and that is an absolutely wonderful, lucky, awesome feeling. [Patient, male, ].

From the perspective of formal caregivers, emergency planning was not only needed to help patients and informal caregivers better deal with exacerbations, but also to avoid unnecessary hospitalisations and emergency measures that did not necessarily correspond to the patients’ best interest, such as intubation. In their experience, persons with advanced COPD were often stuck in a vicious cycle of repeated emergency hospital admissions that negatively impacted their quality of life and that of their informal caregivers. Thus, formal caregivers, especially PC physicians and pulmonologists with experience in intensive care units, remarked the need for early advance care planning directives (ACP) and PC for persons with advanced COPD. They also mentioned that emergency medication, instructions on how to handle exacerbations, as well as contact persons for emergencies could also help manage dyspnoea and anxiety and reduce avoidable hospitalisations (ST3: Q1).

#### Psychosocial care

Besides dyspnoea and anxiety, negative feelings and limited social interactions were considered burdensome. Some persons described the psychological burden of COPD to be as bad as the physical symptoms for patients, and to constitute an additional factor that informal caregivers have to take care for (ST1: Q13).

Further, informal caregivers carried themselves a psychosocial burden in the context of caring for the persons with advanced COPD (ST2: Q10). Being responsible for the patients’ wellbeing was described from being difficult to being a major burden; some informal caregivers prioritized the patient’s needs at the cost of their own. They also reported negative feelings of loss, longing and sadness, frustration, and to be troubled by thoughts about the nearing death of the person with advanced COPD (ST2: Q5). Caring for a person with advanced COPD significantly affected informal caregivers’ social lives and sense of wellbeing:

I try, at least, to do something for myself two or three times a week by going to the gym. But I don’t really stay away much longer than that. I try to / well, yes, I miss a lot, actually. That I can’t go out and be around people the way I used to. […] But he doesn’t want that anymore. He doesn’t want to be left alone now. And putting him in a care home, that’s not something I would do. That’s just not me. Yes, I really do miss a lot. I’d rather be away more often. We used to travel so much before the illness got worse. And I miss all of that terribly. Having so many people around us. I really miss that. I just can’t talk about it, because whenever I do, I start to cry. So better not / yeah. [Informal caregiver, female]

When directly asked about the need for formal psychosocial support, only few expressed need for professional support (ST1: Q18). Most persons with advanced COPD or their informal caregivers often claimed not to currently have a need for it, or that the need is met by own personal resources such as friends or family members, even though they were aware that this weighed on their supporters. Some informal caregivers considered themselves to be the emotional support for the person with advanced COPD, for example an informal caregiver who discussed sensitive matters related the end-of-life preparation with her mother (ST2: Q6). The rejection of formal psychosocial support in spite of need was apparent in some of the interviews. An informal caregiver explained that his father was clearly depressed but refused to get professional help (ST2: Q7). A person with advanced COPD narrated:

I had actually forgotten about it for two years, but the last time I went to my GP, I talked with him about the fact that I can’t go on living like this. I needed… I think I needed some psychological help, and he gave me an address. I even stood there at the door with my walker to make the appointment. But I didn’t go in, because I thought to myself, “You can handle this on your own.” Yeah. […] I didn’t actually use it, no. I did get psychiatric help when I was at rehab. But that… well, it really wasn’t what I had hoped for. […] [Patient, male].

Some formal caregivers perceived the communication with persons with advanced COPD as difficult, mentioning that they perceived a sense of shame related to the disease, indolence, and a lack of acceptance and avoidance from the patient’s side to discuss the disease or its prognosis (ST3: Q12, Q13). They also observed lacking motivation to utilize psychosocial services and other healthcare services in case of existing depression, and that psychological support services, among others, were not sufficiently available in Germany. Further, formal caregivers expressed that to facilitate the provision of psychosocial support for persons with advanced COPD, it was necessary to get the informal caregivers on board, and to improve case management within the healthcare system (ST3: Q17).

As for the informal caregivers, care work often translated in limited or lack of time for leisure and social activities, which constituted a psychosocial burden but was also described as a barrier to access psychosocial support services such as self-help groups (ST2: Q8):

I have information [about self-help groups], but I can’t go there anyway, because I can’t leave my husband alone. That doesn’t help me, right? If I go there, I can’t just stay for half an hour either. You’re there longer at these meetings. Yes, and what am I supposed to do with my husband during that time? (…) Basically, it’s really only for people who can leave the sick person at home alone. [Informal caregiver, female]

Formal caregivers underscored an observed need for psychological and psychiatric care among persons with advanced COPD and their informal caregivers. They emphasized that these services should be provided to patients in the context of home-based healthcare (ST3: Q11). However, they also highlighted that adequate services to fulfil these needs are lacking due to long waiting times for psychotherapy treatment, or because psychotherapists and psychiatrists do not pay home visits.

An informal caregiver with a medical background suggested that PC could be helpful for a person with advanced COPD who, at the time of the interview, was not receiving it yet. She explained that the emergency medications and psychosocial services involving the family offered by PC could help alleviate their suffering. Indeed, informal caregivers receiving PC reported some degree of psychosocial relief, for example in the form of a sense of security and reassurance, as previously presented.

#### Organizational support

Persons with advanced COPD usually required assistance to manage their everyday lives. This assistance was provided to them by informal caregivers, nursing care teams, and housekeeping services (ST1: Q3, Q4, Q14). The support of persons with advanced COPD rested sometimes on only one informal caregiver, often the spouse or a child. Formal caregivers considered single persons with advanced COPD to be particularly in need of care services and social support. They observed that underlying mental health issues prevented persons with COPD from accessing such services and made them harder to approach (ST3: Q13, Q17).

Informal caregivers were burdened by care for the patients, household chores, and organizational and bureaucratic activities related to healthcare provision for the patient (ST2: Q6, Q11). These included dealing with healthcare insurance claims, the organization of nursing support, ordering and picking up prescriptions, dealing with healthcare and supply providers, accompanying patients to medical appointments, taking care of the availability and functioning of medical devices, and being contact persons for treating physicians and other medical staff. Also, some informal caregivers helped patients get advance care planning directives (ST2: Q6).

Obtaining information about formal organizational support was reported to be time consuming; information about insurance claims, potential disability benefits or early retirement options was not always made available by formal caregivers. Accordingly, several persons expressed to have received this kind of information from relatives or acquaintances working in related areas. It was mentioned that the decision-making process on such matters was not always transparent enough, and some informal caregivers expressed a sense of injustice regarding the allocation of support by health insurance companies or governmental social services.

Supporting a person with advanced COPD left little time to informal caregivers for themselves (ST2: Q8). Some expressed the need of support in caring for the patient, so that they could temporarily leave home to fulfil own needs, such as to attend medical or other appointments, or to take rest from care work (ST2: Q12). An informal caregiver formally registered as caring person expressed how being overwhelmed with care and household responsibilities prevented her from organising and taking a needed break:

Yes, I also have 20 days of vacation per year. And I can also / during that time someone, like a friend or acquaintance, could come and take care of my husband. But until now, I’ve never done that. For the past three years, I haven’t taken that time. And he (laughs), he said, “You’ve saved the insurance company money!” (laughs) Because I didn’t use it, and of course, I COULDN’T use it. […] Because it was all just too much […] he was constantly in the hospital. And everything came at once. […] I didn’t even have the space / or the time to organize anything, like who could come. A friend, or a friend of my daughter’s, or / I just had no time and no energy left. I only wanted to just get through things with him / and then finally rest, sleep, you know. So I didn’t do it, unfortunately. [Informal caregiver, female]

While nursing care was considered helpful in supporting informal caregivers, this support was not always considered sufficient (ST2: Q11). The same applied to housekeeping services: the two hours a week covered by the health insurance were considered too little to provide substantial relief to informal caregivers, and some paid out of pocket for more hours.

Some GPs and pulmonologists recognized that providing psychosocial and organizational support to informal caregivers was part of their role (ST3: Q19), while others preferred to put the patient in the forefront instead. The organizational support provided by PC teams to patients and informal caregivers was considered very helpful by recipients (ST4: Q7). An informal caregiver explained that a PC team organized a volunteer to accompany her mother (i.e., the patient). With this support, her father could go out and have some time by himself (ST4: Q5). A person with advanced COPD said to have benefited from the organizational support provided by the hospital social services and the PC team. They arranged a bathtub lift and other aids for him in an uncomplicated manner (ST4: Q4). Obtaining advanced care directives was considered particularly difficult, and assistance from PC teams to inform and complete the forms with the patients was reported to be relieving for both patients and informal caregivers (ST4: Q6).

#### Timely information about COPD and palliative care

Persons with advanced COPD and informal caregivers were caught by surprise by the severity of symptoms in the advanced stage of the disease and claimed to have not been aware of how limiting the condition could become (ST1: Q7, Q16, Q17; ST2: Q9). We identified the need for earlier information about COPD’s progression and what it means to live with advanced COPD.

Information about COPD’s progression and potential lethality of COPD was not consistently communicated. As a result, persons with COPD failed to grasp the possible serious implications of COPD until they started experiencing them first hand. This was perceived to be partly because knowledge about COPD is not widespread among the general population in contrast to other conditions (e.g., lung cancer).

An informal caregiver explained that, at the time she was confronted with the advanced stage, she was overwhelmed and unable to process such information. Obtaining this information earlier could have been helpful to prepare for the later stages of the disease (ST2: Q13). Some claimed not to have received enough or clear information from formal caregivers about what COPD is and how it can develop over time. There was a perceived general lack of knowledge about the progression of COPD among patients:

You don’t notice how serious it can become. Right, so I’ve only recently realized how bad it can get. Because before, I still felt relatively well. […] Many people, they know the basic term, they know that something has been detected inside. But to actually catch someone and say: “Hey, listen. You have to prepare yourself for this and that in life now, for what’s coming,” and THEN that you might need oxygen at some point. Or that you’ll have to go to the hospital more often because the […] exacerbations get worse. All of that, that’s really my concern, right? That people who are affected by this are at least told: “Do you know what’s going to come your way with this disease?” But no one tells them that. […] So, all this information as early as possible, because it’s a deadly disease. Yes. They shouldn’t hold back, they should say it is a deadly disease. Whether you have lung cancer or COPD, both lead to death. [Patient, female].

Some persons searched for information about COPD on their own or said that such information was hard to find (ST2: Q15). There was a general perception that health insurances, formal caregivers or the media should provide more information about COPD to better inform the public, also in connection to smoking prevention (ST1: 16). Some persons with advanced COPD mentioned that they were early informed about the progression by formal caregivers, but that they did not listen because they were feeling alright at that time. Some said they would have done things differently (e.g., quitting smoking earlier) if they had taken the disease more seriously or been better informed about exposure risks (ST1: Q17).

The need for early information about the nature and progression of COPD as well as about possibilities to access psychosocial and organizational support was also recognized by formal caregivers (ST3: Q14, Q15, Q17). They expressed that persons in need of such information are not sufficiently receptive to it when they are at an advanced stage and start receiving PC (ST3: Q15). However, formal caregivers also reported that communication about the disease and progression was particularly challenging with persons with COPD, especially when compared with patients with lung cancer (ST3: Q12). Persons with COPD were described by formal caregivers as less compliant with medical advice or less likely to make use of healthcare services, such as smoking cessation seminars or lung sports. Formal caregivers mentioned several potential reasons for challenging communication: a lack understanding and acceptance about COPD, shame and a sense of guilt, as well as dyspnoea making the following of difficult conversations hard, and patients not wanting to talk about the disease or being very difficult to approach (ST3: Q12, Q16). Some formal caregivers shared that they do not provide information to patients about the lethality of COPD or related comorbidities, nor about palliative care options. They alluded to a lack of time as the main reason.

By contrast, having a trusting patient-physician relationship, making home visits and involving informal caregivers were described to facilitate communication and informational flow between formal caregivers and persons with advanced COPD (ST3: Q13). Patients who described the relationship with their treating physicians in a positive way explained that they could talk about everything with them, and to feel well informed about COPD.

When asked if they think they could benefit from PC, persons with advanced COPD showed wariness or resistance to discuss the topic, saying for example that the topic is not relevant for them yet. Formal caregivers also expressed that some persons with advanced COPD and informal caregivers explicitly refused conversations related to PC, while others were reported to be thankful for such an honest exchange. According to formal caregivers, PC could first be introduced in the conversation in terms of its potential benefits for symptom relief (ST3: Q16, Q18).

In contrast, persons who had received PC were open to talk about it and felt that PC had been very beneficial. An informal caregiver expressed that PC was the best form of care they experienced during the whole disease trajectory, but that she did not know that persons with COPD were eligible for PC until a physician in a specialized clinic suggested it (ST4: Q8). She also mentioned that she did not think her mother would have been open for PC much earlier:

*Because even today, with the palliative care, my mother still has the thought: maybe I will live another two or three years. She does not accept the approaching end. Which I do not want to judge; I can understand that very well. I mean, who wants to accept that they won’t live much longer?* [Informal caregiver, female].

## Discussion

Based on interviews with patients, informal caregivers and formal caregivers, we identified the following main domains of unmet needs among persons with advanced COPD and their informal caregivers: (1) management of dyspnoea and anxiety, (2) home-based healthcare, (3) emergency planning and support, (4) psychosocial care, (5) organizational support, and (6) timely information about COPD and PC. Persons who had received PC in the context of advanced COPD and their informal caregivers expressed relief and fulfilment of these needs. Although patients reported low EQ-5D-VAS scores and high anxiety and depression levels, most reported to feel adequately supported. We may explain this discrepancy with Bradshaw’s theoretical assumption of needs: namely that needs may not be recognized by potential beneficiaries when there is a lack of awareness of the services that may fulfil them, or that needs may only be recognized once they are fulfilled [31]. Further, it is possible that patients evaluated the support provided by their informal caregivers. In the following, we discuss how these needs could be addressed, and the potential role of PC in doing so.

### (Non-) Pharmacological therapies and training to manage dyspnoea and anxiety

In line with previous publications [6, 12, 45–47], our study showed that dyspnoea and anxiety were considered the most burdensome symptoms for persons with advanced COPD, and that their informal caregivers shared the experience of breathlessness [13]. This underscores the need for and importance of managing these symptoms. We further identified an unmet need for training and information regarding the use of inhalers and non-pharmacological therapies to better manage dyspnoea and anxiety, including breathing techniques (such as pursed-lip breathing). Such forms of training have been shown to decrease the severity of breathlessness and to improve quality of life [48–50]. Also, pulmonary rehabilitation is considered helpful, especially after hospitalizations [48, 51] and it is recommended besides respiratory physiotherapy by the German Clinical Practice Guideline for COPD [28]. However, our findings showed that access to such training or therapies remained limited for persons with advanced COPD because of their restricted mobility and the lack or therapists doing home visits, or because physicians did not have their prescription on their radar. We suggest that such training and therapies should be consistently made available to persons with COPD at earlier stages. This would help to ensure that they are sufficiently familiar with the techniques when these become increasingly necessary, e.g., in case of exacerbations or shortage of medicines. Non-pharmacological techniques and psychoeducation focusing on anxiety-management could also help ease the burden of worry about future exacerbations among persons with advanced COPD, as discussed below under psychosocial support. Research has shown that distress caused by dyspnoea may also be due to a lack of training of informal caregivers [13]. Informal caregivers should therefore also receive such trainings to enable them to assist persons with advanced COPD in case of dyspnoea. This could also help fulfil the need to be better prepared for emergency situations, as also informal caregivers play an active role in the management of dyspnoea and anxiety [13].

Psychosocial support was identified as a need, but it may also play a role in managing dyspnoea and anxiety (see section on psychosocial services). Medications such as benzodiazepines and opioids were also considered helpful to manage anxiety and dyspnoea, respectively. However, reported reservations against their prescription align with previous studies [8]. Further, these therapies are not explicitly mentioned in the German Clinical Practice Guideline for COPD [28]. While it is important to mention that some studies report a lack of evidence regarding the effectiveness of opioid prescription to manage dyspnoea and anxiety for COPD, they also suggest that persons with advanced COPD do not have many therapy options left [53]. Integrating PC in the healthcare of persons with COPD as the burden of dyspnoea and anxiety becomes harder to manage could help provide safer access to these medications.

### Improving home-based healthcare and emergency management

Our analysis suggested that the need for home-based healthcare was significant among persons with advanced COPD. However, this need remained largely unmet unless specialized PC was received. In Germany, GPs routinely conduct home visits, but the total number of visits (and GPs) has declined in past years [54, 55]. Pulmonologists and other specialists do not conduct home visits in general. Because access to physicians’ offices was difficult or no longer possible, many persons with advanced COPD in our study no longer received outpatient healthcare from pulmonologists or even GPs. They relied on emergency services for healthcare provision instead. This was not only challenging and distressing for patients and informal caregivers but represents also a challenge for the healthcare system and society due to the significant cost of avoidable hospitalizations of persons with COPD [56]. Home-based healthcare is an integral part of SOPC in Germany, and persons who received it reported relief and an increased sense of security in our study. However, it is only provided to patients with a complex symptom burden and, thus, cannot fill the home-based healthcare delivery gap for all persons with advanced COPD. To improve home-based healthcare, formal and informal caregivers in our study suggested sending non-physician medical staff to conduct home visits and assist GPs, to increase the use of telemedicine, and to promote video-consultation appointments with psychotherapists. Further innovative concepts such Hospital@Home, which aim to deliver hospital-level care in home-based settings, could prove favourable in lowering rehospitalisation rates for persons with COPD and in providing positive clinical and cost-effective outcomes [57]. However, while promising, such concepts and structures are still developing in Germany and have not been implemented nationwide yet [57, 58].

Reducing avoidable hospitalizations also goes hand in hand with the need for better emergency planning among persons with advanced COPD and informal caregivers. Individualized breathlessness plans, including the use of pharmacological and non-pharmacological strategies, has been shown to be an acceptable and useful intervention [53]. A study on PC needs emphasized the need for person-centred approaches to manage exacerbations, as well as access around the clock, and supported decision making for informal caregivers [24], while a Cochrane meta-analysis further showed that exacerbation action plans reduce hospitalization rates [59]. PC teams may fulfil this need, as they were described by our participants as reliable and effective in providing a sense of security to handle emergencies to persons with COPD and informal caregivers. However, we did not examine specific clinical criteria for escalation from home-based palliative care to emergency hospitalization. Therefore, reports of successful symptom management at home should not be interpreted as replacing standard clinical assessment of acute exacerbations. Decisions regarding hospitalization remain individualized and according to clinical guidelines should consider symptom severity, clinical stability and also patient preferences.

An early discussion of advanced care planning (ACP) may also be beneficial for persons with COPD and informal caregivers, as our findings showed that dealing with ACP directives in the advanced stage of the diseases could be overwhelming for them. The European Respiratory Society recommends to periodically revise ACP, while respecting the needs and values of persons with COPD and their caregivers [22]. However, an effective patient-clinician communication is needed, as the disease trajectory of COPD makes it very difficult to predict the best time for initiating end-of-life care [60]. Further, some persons are reluctant to make fixed decisions about the future [61] or, as presented here, are reluctant to discuss matters related to death. As part of its holistic approach, in Germany ACP was also facilitated by PC teams, from which some persons in our study said to have benefited.

### Lowering the threshold of psychosocial and organizational support services

Needs regarding the management of feelings, worries, as well as anxiety and depression among persons with COPD have been previously identified [15], and depression and anxiety are recognized as common comorbidities of COPD [62]. It has been suggested that less than one third of the persons with COPD affected by depression or anxiety symptoms receive appropriate treatment [62]. Reasons include patient perceived barriers, lack of compliance, lack of a standardized diagnostic approaches, and scarcity of adequate treatment resources [62]. Like in other studies [15], our participants did not report spiritual needs, even though we explicitly asked.

Informal caregivers may also experience depression and anxiety symptoms when they care for a person with advanced COPD [63]. The number of caregiving tasks may predict negative mental health outcomes in the wives caring of their husbands with COPD [64]. However, while the importance of the role of informal caregivers was widely recognized by formal caregivers in our study, their psychosocial needs often remained “hidden” [12]. Putting the needs of persons with COPD first and being overwhelmed with caring tasks constituted further potential barriers to access psychosocial support in our findings.

Our study found that the need for psychosocial support for persons with advanced COPD and their informal caregivers was recognized by formal caregivers. This need was, however, rarely expressed by persons with advanced COPD or their informal caregivers, even when they appeared to be extremely taxed by the experience of living with advanced COPD or caring. This finding not only shows that normative needs may diverge from the perceived needs of patients and informal caregivers, but also highlights the need for a consistent screening for psychological and psychiatric conditions and for low-threshold psychosocial support services that consider reservations against professional support for mental health issues even when they may be needed. Studies suggest that non-pharmacological therapies such as pulmonary rehabilitation, a collaborative care model, and psychological therapy (e.g., cognitive behavioral therapy) may be helpful reducing depression and anxiety symptoms among persons with COPD [62, 65, 66]. Informal caregivers receiving PC expressed to benefit from the support and guidance of PC teams, and from organizational support provided by them.

Persons with advanced COPD may be entitled to a series of benefits in form of financial support or assistive devices provided by the statutory health insurance or social authorities in Germany. However, not all informal caregivers were well informed about the support they could apply for. Navigating German bureaucracy was considered draining and time consuming by our participants. Since persons with advanced COPD rarely regularly accessed outpatient care, they often received social services though hospitals. It is conceivable that it is more beneficial to receive information about organizational support before having an acute need for it. GPs and pulmonologists could inform persons with COPD about the possibility of making certain claims, to assist with applications they are responsible for, or to refer to social services that can support patients and informal caregivers. However, not all formal caregivers in our study saw providing psychosocial and organizational support as part of their role. Thus, increasing awareness about their role as facilitators seems also important to improve access to organizational support services.

To be accessed, it is crucial that psychosocial and organizational support services consider the limited mobility of persons with advanced COPD and their informal caregivers. GPs or medical assistants making home visits could better assess needs of persons with advanced COPD during visits and provide support on the spot if required. Multidisciplinary PC teams played an important role in providing accessible psychosocial and organizational support to some study participants, for example by taking care of assessing organizational needs or by providing practical support though volunteer helpers. However, these teams provided SOPC, which is not always indicated for persons with advanced COPD. Thus, there is a need to improve access to psychosocial and organizational support services within the context of regular healthcare. This, however, may require structural changes in the German healthcare system, as mentioned by some physicians in our study. We acknowledge that our suggestions may be hard to implement in the current context of the German healthcare system, and that a review of their feasibility may be necessary.

### Early and sensible communication about COPD’s progression and PC

Numerous studies have reported that persons with COPD do not receive enough information regarding the disease’s progression and prognosis, constituting an important unmet need [5, 15, 61, 67]. Our study supports these findings, adding that this information gap may be particularly salient among persons who do not have a close relationship with their GP or pulmonologist. Indeed, some formal caregivers shared that they do not inform persons with COPD about its potential lethality.

In view of these findings, it is relevant to emphasize the importance of a transparent communication between patients and formal caregivers, and of seeing the communication of progression and prognosis as an ongoing process [68]. A reason why persons with COPD have limited access to PC is because formal caregivers do not talk about it [69]. Even though our results suggest that persons with advanced COPD and informal caregivers benefit greatly from PC, persons who were not receiving PC yet had reservations about discussing how it could help them. Such reservations may further hinder access to PC. To increase acceptance and improve access to PC, early and patient-centred communication about the progression of COPD could help. Further, information about what PC can do to mitigate symptoms and support persons with advanced COPD and informal caregivers could be useful. A review recommends that formal caregivers start discussions about PC early in the disease trajectory, and not during critical situations or when PC has become necessary [70]. Informational discussions on PC can be initiated in view of specific triggers, such as the need for ventilation during the previous twelve months, or observed deterioration of the patient’s condition, to mention a few [70]. Providing such information would require skills and resources, which in the German context could be made available, for example, through the disease management programme for COPD (DMP COPD). Further, persons with COPD could profit from greater general awareness in society about the disease, as it was often claimed that while lung cancer is well known, most persons do not understand what it means to live with advanced COPD. Further research is needed to explore and develop possible interventions targeting these areas.

### Strengths

The multi-perspective approach of this study allowed us to capture needs and the role of PC, to identify gaps and overlapping between patient-centred and expert-centred perspectives, and to capture the realities of healthcare provided to persons with advanced COPD by GPs, pulmonologists, and PC physicians. Further, the number of analyzed interviews (*n* = 53) allowed us to reach thematic saturation even though the sample of patients and informal caregivers was well mixed in terms of educational level, place of living and ethnicity. By including persons who had received PC and those who had not, we were able to identify comparable needs [31] that we may have overlooked if we had only considered those who had not received PC. Finally, our recruiting strategy was designed to maximize the chances that patients meet our definition of advanced COPD, and also to recruit persons informally caring of persons meeting that criteria.

### Limitations

Recruitment of patients relied on their physicians. We therefore relied on the information provided by physicians regarding inclusion criteria (e.g., the COPD diagnosis was not confirmed as part of this study). Also, we cannot rule out that physicians applied covert selection processes, for instance, refraining from contacting patients that were considered too sick to participate. Recruiting physicians received a monetary incentive. Although this approach may have introduced a risk of selection bias, it was deemed necessary given the anticipated challenges in identifying patients who met the inclusion criteria and were also able to participate in the study. However, we cannot rule out selection bias, as it is possible for example that persons with more adverse views toward PC were less likely to participate. Our study only considered specialist physicians as formal caregivers and did not include the perspective of other professions formally involved in the healthcare provision of persons with advanced COPD and who may have more regular contact with them and their informal caregivers (e.g., medical assistants, nurses, care nurses, etc.). Although the needs identified in our study overlap with findings from the international literature, the transferability of our proposed suggestions beyond the German healthcare system may be limited due to possible differences regarding structural barriers towards PC access or home-based care, as the availability of PC structures and services varies across the world [71]. Finally, findings of this study were not returned to participants for validation. To enhance transparency and allow readers to assess the credibility of our interpretations, we provided verbatim quotations in both the main text and in the Supplementary Tables 1 to 4. This enables readers to evaluate whether the identified topics represent plausible interpretations grounded in the data rather than mere researcher projections. This is particularly important for implicit needs, which, by their nature, may be difficult for participants to validate because they are not always consciously recognized.

## Conclusions

Persons with advanced COPD and their informal caregivers face numerous burdens and have manifold unmet healthcare needs. Persons who had access to PC benefited from (non-) pharmacological therapies, a holistic approach involving and supporting informal caregivers, preparation for emergencies and home-based healthcare through emergency medications, emergency plans, and an emergency contact number, as well as regular home visits by PC teams. Also, they claimed to benefit from the multi-professional support given to address psychosocial and organizational needs. However, PC remains largely underused among persons with advanced COPD in Germany. Providing COPD patients and their informal caregivers with early information about COPD and its progression, as well as about PC, could help raise awareness and acceptance of PC. This could encourage earlier use of PC in patients with advanced COPD as soon as indicated. However, more research is required to generate more specific action recommendations to specialized physicians to improve the (palliative) care of people with advanced COPD and their informal caregivers.

## Supplementary information

Below is the link to the electronic supplementary material.

Supplementary Material 1: Additional file 1: Table 3 – Spontaneous thoughts when hearing “palliative care” among persons with advanced COPD and informal caregivers who had not received PC yet.

Supplementary Material 2: Additional file 2: Table 4 – Spontaneous thoughts when hearing “palliative care” among persons with advanced COPD and informal caregivers who were currently receiving or had received PC.

Supplementary Material 3: Additional file 3: Table 5 - Barriers to access palliative care (PC) for persons with advanced chronic obstructive pulmonary disease (COPD) and their informal caregivers, verbatim quotes from formal caregivers, listed according to categories of the coding scheme.

Supplementary Material 4: Additional file 4: Table 6 – Suggestions to improve access to PC for persons with advanced COPD and their informal caregivers, verbatim quotes from formal caregivers, listed according to categories of the coding scheme.

Supplementary Material 5: Additional file 5: Interview guide for patients

Supplementary Material 6: Additional file 6: Interview guide for informal caregivers

Supplementary Material 7: Additional file 7: Interview guide for general practitioners and pulmonologists

Supplementary Material 8: Additional file 8: Interview guide for palliative care physicians

## Data Availability

The data that support the findings of this study are available from the corresponding author upon reasonable request, subject to compliance with data protection regulations.

## References

[1] de Oca MM, Perez-Padilla R, Celli B, Aaron SD, Wehrmeister FC, Amaral AFS, et al. The global burden of COPD: epidemiology and effect of prevention strategies. The Lancet Respiratory Medicine. Elsevier Ltd; 2025. pp. 709–24. 10.1016/S2213-2600(. 24)00339-4 PubMed PMID: 40684784. 40684784

[2] Vos T, Lim SS, Abbafati C, Abbas KM, Abbasi M, Abbasifard M, et al. Global burden of 369 diseases and injuries in 204 countries and territories, 1990–2019: a systematic analysis for the Global Burden of Disease Study 2019. Lancet. 2020;396(10258):1204–22. 10.1016/S0140-6736(20)30925-9. PMC7567026 33069326

[3] Kahnert K, Jörres RA, Behr J, Welte T. The Diagnosis and Treatment of COPD and Its Comorbidities. Dtsch Arztebl Int. 2023;120:434–44. 10.3238/arztebl.m2023.027. PMC10478768 36794439

[4] Global Initiative for Chronic Obstructive Lung Disease (GOLD). GOLD Report 2026 [Internet]. 2025 [cited 2025 Dec 16]. Available from: https://goldcopd.org/2026-gold-report-and-pocket-guide/

[5] Seamark D, Blake S, Seamark C, Halpin D. Living with severe chronic obstructive pulmonary disease (COPD): perceptions of patients and their carers. An interpretative phenomenological analysis. Palliat Med. 2004;18:619–25. 10.1191/0269216304pm928oa 15540670

[6] Gardiner C, Gott M, Payne S, Small N, Barnes S, Halpin D, et al. Exploring the care needs of patients with advanced COPD: An overview of the literature. Respir Med. 2010;159–65. 10.1016/j.rmed.2009.09.007 . PubMed PMID: 19818590. 19818590

[7] Blinderman CD, Homel P, Andrew Billings J, Tennstedt S, Portenoy RK. Symptom Distress and Quality of Life in Patients with Advanced Chronic Obstructive Pulmonary Disease. J Pain Symptom Manage. 2009;38(1):115–23. 10.1016/j.jpainsymman.2008.07.006 . PubMed PMID: 19232893. 19232893

[8] Schroedl CJ, Yount SE, Szmuilowicz E, Hutchison PJ, Rosenberg SR, Kalhan R. A qualitative study of unmet healthcare needs in chronic obstructive pulmonary disease: A potential role for specialist palliative care? Ann Am Thorac Soc. 2014;11(9):1433–8. 10.1513/AnnalsATS.201404. -155BC PubMed PMID: 25302521. PMC4298987 25302521

[9] Ek K, Ternestedt BM. Living with chronic obstructive pulmonary disease at the end of life: a phenomenological study. J Adv Nurs. 2008;4(62):470–8. 10.1111/j.1365-2648.2008.04611.x 18373613

[10] Guthrie SJ, Hill KM, Muers MF. Living with severe COPD. A qualitative exploration of the experience of patients in Leeds. Respir Med. 2001;95(3):196–204. 10.1053/rmed.2000.1021 . PubMed PMID: 11266237. 11266237

[11] Gore JM, Brophy CJ, Greenstone MA. How well do we care for patients with end stage chronic obstructive pulmonary disease (COPD)? A comparison of palliative care and quality of life in COPD and lung cancer. Thorax. 2000;55(12):1000–6. 10.1136/thorax.55.12.1000 . PubMed PMID: 11083884. PMC1745647 11083884

[12] Bergs D. The Hidden Client - Women caring for husbands with COPD: Their experience of quality of life. J Clin Nurs. 2002;11(5):613–21. 10.1046/j.1365-2702.2002. 00651.x PubMed PMID: 12201888. 12201888

[13] Ferreira DH, Kochovska S, Honson A, Phillips JL, Currow DC. Two faces of the same coin: A qualitative study of patients’ and carers’ coexistence with chronic breathlessness associated with chronic obstructive pulmonary disease (COPD). BMC Palliat Care. 2020;19(1). 10.1186/s12904-020-00572-7 . PubMed PMID: 32375747. PMC7203967 32375747

[14] Bove DG, Zakrisson AB, Midtgaard J, Lomborg K, Overgaard D. Undefined and unpredictable responsibility: A focus group study of the experiences of informal caregiver spouses of patients with severe COPD. J Clin Nurs. 2016;25(3–4):483–93. 10.1111/jocn.13076 . PubMed PMID: 26818373. 26818373

[15] Gardener AC, Ewing G, Kuhn I, Farquhar M. Support needs of patients with COPD: A systematic literature search and narrative review. International Journal of COPD. Dove Medical Press Ltd.; 2018. pp. 1021–35. 10.2147/COPD.S155622 PubMed PMID: 29628760. 10.2147/COPD.S155622 PMC5877489 29628760

[16] Sigurgeirsdottir J, Halldorsdottir S, Arnardottir RH, Gudmundsson G, Bjornsson EH. COPD patients’ experiences, self-reported needs, and needs-driven strategies to cope with self-management. Int J COPD. 2019;14:1033–43. 10.2147. /COPD.S201068 PubMed PMID: 31190788. 10.2147/COPD.S201068 PMC6529673 31190788

[17] Jones I, Kirby A, Ormiston P, Loomba Y, Chan KK, Rout J, et al. The needs of patients dying of chronic obstructive pulmonary disease in the community. Fam Pract. 2004;21(3):310–3. 10.1093/fampra/cmh317 . PubMed PMID: 15128695. 15128695

[18] Sami R, Salehi K, Akbari M, Zolfaghari M, Atashi V, Riyahi A. The needs of the family caregivers of patients with end-stage chronic obstructive pulmonary disease: a qualitative study in Iran. BMC Nurs. 2025;24(1). 10.1186/s12912-025-03871-7. PMC12465522 41013527

[19] Skilbeck J, Mott L, Page H, Smith D, Hjelmeland-Ahmedzai S, Clark D. Palliative care in chronic obstructive airways disease: a needs assessment. Palliat Med. 1998;12(4) : 245–54 10.1191/02692169867712462 9743823

[20] Buckingham S, Kendall M, Ferguson S, Macnee W, Sheikh A, White P, et al. HELPing older people with very severe chronic obstructive pulmonary disease (HELP-COPD): Mixed-method feasibility pilot randomised controlled trial of a novel intervention. NPJ Prim Care Respir Med. 2015;25. 10.1038/npjpcrm.2015.20 . PubMed PMID: 26028347. PMC4532154 26028347

[21] World Health Organization. Palliative Care [Internet]. 2020 [cited 2025 Nov 7]. Available from: https://www.who.int/news-room/fact-sheets/detail/palliative-care

[22] Janssen DJA, Bajwah S, Boon MH, Coleman C, Currow DC, Devillers A, et al. European Respiratory Society clinical practice guideline: palliative care for people with COPD or interstitial lung disease. Eur Respir J. 2023;62(2). 10.1183/13993003.02014-2022 . PubMed PMID: 37290789. 37290789

[23] Crawford GB, Price SD. Team working: Palliative care as a model of interdisciplinary practice. Medical Journal of Australia. Australasian Medical Publishing Co. Ltd; 2003. 10.5694/j.1326-5377.2003.tb. 05575.x PubMed PMID: 12964934. 12964934

[24] Fu Y, Mason A, Boland AC, Linklater G, Dimitrova V, Doñate-Martínez A, et al. Palliative Care Needs and Integration of Palliative Care Support in COPD: A Qualitative Study. Chest. 2021;159(6):2222–32. 10.1016/j.chest. .2020.12.055 PubMed PMID: 33434498. 33434498

[25] Vermylen JH, Szmuilowicz E, Kalhan R. Palliative care in COPD: An unmet area for quality improvement. International Journal of COPD. Dove Medical Press Ltd.; 2015. pp. 1543–51. 10.2147/COPD.S74641 PubMed PMID: 26345486. 10.2147/COPD.S74641 PMC4531041 26345486

[26] Zornitzki L, Sror N, Bar-Shai A, Tellem R, Banai S, Frydman S et al. Underutilization of palliative care in advanced COPD and heart failure: associations, disparities, and the role of specialists. Ther Adv Respir Dis. 2025;19 (1-10). doi:10.1177/17534666251364056 PubMed PMID: 40796380. 10.1177/17534666251364056 PMC12344236 40796380

[27] --Autoren L, Kahnert K, Götschke J, Bausewein C, Behr J. Die palliative Versorgung von COPD-Patienten [Internet]. Available from: www.springermedizin.de/kurse-mmw 10.1007/s15006-020-0016-8 32514988

[28] Kassenärztliche Bundesvereinigung B, Träger. Nationale VersorgungsLeitlinie COPD Teilpublikation der Langfassung 2. Auflage, Version 1 [Internet]. 2021. Available from: www.leitlinien.de.

[29] Wu YP, Thompson D, Aroian KJ, Mcquaid EL, Deatrick JA, Commentary. Writing and evaluating qualitative research reports. Journal of Pediatric Psychology. Oxford University Press; 2016. pp. 493–505. 10.1093/jpepsy/jsw032 . PubMed PMID: 27118271. PMC4888120 27118271

[30] Batzler YN, Schallenburger M, Schwartz J, Marazia C, Neukirchen M. The General Public and Young Adults’ Knowledge and Perception of Palliative Care: A Systematic Review. Healthcare (Switzerland). Multidisciplinary Digital Publishing Institute (MDPI); 2024. 10.3390/healthcare12100957. PMC11121430 38786369

[31] Bradshaw J. A taxonomy of social need. In: McLachlan G, editor. Problems and progress in medical care: essays on current research [Internet]. London: Oxford UNiversity Press; 1972. pp. 71–82. Available from: https://eprints.whiterose.ac.uk/

[32] Tong A, Sainsbury P, Craig J. Consolidated criteria for reporting qualitative research (COREQ): A 32-item checklist for interviews and focus groups. Int J Qual Health Care. 2007;19(6):349–57. 10.1093/intqhc/mzm042 . PubMed PMID: 17872937. 17872937

[33] Barnett M. Chronic obstructive pulmonary disease: a phenomenological study of patients’ experiences. J Clin Nurs. 2004; 14(7):805–12. 10.1111/j.1365-2702.2005.01125.x 16000094

[34] Ek K, Andershed B, Sahlberg-Blom E, Ternestedt BM. The unpredictable death - The last year of life for patients with advanced COPD: Relatives’ stories. Palliat Support Care. 2015;13(5):1213–22. doi:10.1017/S1478951514001151 PubMed PMID: 25315360. 10.1017/S1478951514001151 25315360

[35] Hirakawa Y, Aita K, Nishikawa M, Arai H, Miura H. Facilitating Advance Care Planning for Patients With Severe COPD: A Qualitative Study of Experiences and Perceptions of Community Physicians, Nurses, and Allied Health Professionals [Internet]. Available from: www.homehealthcarenow.org 10.1097/NHH.0000000000000945 PMC7934328 33662966

[36] Scichilone N, Whittamore A, White C, Nudo E, Savella M, Lombardini M. The patient journey in Chronic Obstructive Pulmonary Disease (COPD): a human factors qualitative international study to understand the needs of people living with COPD. BMC Pulm Med. 2023;23(1). 10.1186/s12890-023-02796-8 . PubMed PMID: 38093262. PMC10720133 38093262

[37] Schmidt Stiedenroth K. Reviving A forgotten Sunna: Hijamah (cupping therapy), prophetic medicine, and the re-Islamization of Unani medicine in contemporary India. Contemp Islam. 2019;13(2):183–200. 10.1007/s11562-018-0426-x.

[38] Schmidt-Stiedenroth K, Guthardt L, Genrich M, Köhne M, Stiawa M, Erschens R, et al. What helps hospital staff in times of crisis: qualitative results of a survey on psychosocial resources and stressors in German hospitals during the COVID-19 pandemic. Front Public Health. 2023;11. 10.3389/fpubh.2023.1260079 . PubMed PMID: 37869202. PMC10585258 37869202

[39] Kuckartz U. Qualitative Inhaltsanalyse. Methoden, Praxis, Computer-Unterstützung. Basel: Beltz Juventa; 2018.

[40] Bengtsson M. How to plan and perform a qualitative study using content analysis. NursingPlus Open. 2016;2:8–14. 10.1016/j.npls.2016.01.001.

[41] Guest G, Bunce A, Johnson L. How Many Interviews Are Enough? An Experiment with Data Saturation and Variability. Field methods. 2006;18(1):59–82. 10.1177/1525822X05279903.

[42] Jo YS, Park S, Kim DK, Yoo CG, Lee CH. The cutoff point of clinical chronic obstructive pulmonary disease questionnaire for more symptomatic patients. BMC Pulm Med. 2018;18(1). 10.1186/s12890-018-0601-0 . PubMed PMID: 29482616. PMC5828492 29482616

[43] Plummer F, Manea L, Trepel D, McMillan D. Screening for anxiety disorders with the GAD-7 and GAD-2: A systematic review and diagnostic metaanalysis. Gen Hosp Psychiatry. 2016;39:24–31. .005 PubMed PMID: 26719105. 10.1016/j.genhosppsych.2015.11.005 26719105

[44] Levis B, Sun Y, He C, Wu Y, Krishnan A, Bhandari PM, et al. Accuracy of the PHQ-2 Alone and in Combination With the PHQ-9 for Screening to Detect Major Depression: Systematic Review and Meta-analysis. JAMA. 2020;323(22):2290–300. 10.1001/jama.2020.6504. PMC7284301 32515813

[45] Habraken JM, Willems DL, de Kort SJ, Bindels PJE. Health care needs in end-stage COPD: A structured literature review. Patient Educ Couns. 2007;121–30. 10.1016/j.pec.2007.05.011 . PubMed PMID: 17601696. 17601696

[46] Clari M, Ivziku D, Casciaro R, Matarese M. The Unmet Needs of People with Chronic Obstructive Pulmonary Disease: A Systematic Review of Qualitative Findings. Journal of Chronic Obstructive Pulmonary Disease. COPD: Taylor and Francis Ltd; 2018. pp. 79–88. 10.1080/15412555.2017.1417373 . PubMed PMID: 29308932. 29308932

[47] Farahani MA, Soltani-Arabshahi SK, Kashani M. The needs of family caregivers of patients with chronic obstructive pulmonary disease: a scoping review protocol. BMC Nurs. 2025;24(1):1313. 10.1186/s12912-025-03948-3. PMC12542038 41126154

[48] Burge AT, Gadowski AM, Jones A, Romero L, Smallwood NE, Ekström M, et al. Breathing techniques to reduce symptoms in people with serious respiratory illness: a systematic review. Eur Respiratory Rev. 2024;33(174). 10.1183/16000617.0012-2024 . PubMed PMID: 39477355. PMC11522968 39477355

[49] Ceyhan Y, Tekinsoy Kartin P. The effects of breathing exercises and inhaler training in patients with COPD on the severity of dyspnea and life quality: a randomized controlled trial. Trials. 2022;23(1). 10.1186/s13063-022-06603-3 . PubMed PMID: 36028881. PMC9419340 36028881

[50] Pyszora A, Lewko A. Non-pharmacological Management in Palliative Care for Patients With Advanced COPD. Frontiers in Cardiovascular Medicine. Front Media S A. 2022. 10.3389/fcvm.2022.907664. PMC9339631 35924211

[51] Rochester CL, Alison JA, Carlin B, Jenkins AR, Cox NS, Bauldoff G, et al. Pulmonary Rehabilitation for Adults with Chronic Respiratory Disease An Official American Thoracic Society Clinical Practice Guideline. Am J Respir Crit Care Med. 2023;208(4):E7–26. 10.1164/rccm.202306-1066. ST PubMed PMID: 37581410. PMC10449064 37581410

[52] Kassenärztliche Bundesvereinigung B, Träger. Nationale VersorgungsLeitlinie COPD Kurzfassung 2. Auflage, Version 1 [Internet]. 2021. Available from: www.rki.de/DE/Content/InfAZ/N/Neuartiges_Coronavirus/nCoV.html

[53] Pascoe A, Buchan C, Smallwood N. Provision of palliative care for people with chronic obstructive pulmonary disease: a narrative review. Ann Palliat Med. 2024;13(4):1012–27. 10.21037/apm-24-11. 38902988

[54] Bundesregierung [German Federal Government]. Antwort der Bundesregierung auf die Kleine Anfrage der Abgeordneten Künast u. a. und der Fraktion DIE LINKE: Honorarrückzahlungsforderungen gegen Hausärztinnen und Hausärzte aufgrund von Richtgrößenprüfungen (Drucksache 19/283)[Reply of the Federal Government to the minor interpellation by Members of Parliament Künast et al. and the parliamentary group DIE LINKE: Reimbursement claims against general practitioners due to budgetary audits (Drucksache 19/2683] [Internet]. Berlin et al. 2018 [cited 2025 Nov 21]. Available from: https://dserver.bundestag.de/btd/19/026/1902683.pdf

[55] Voigt K, Taché S, Klement A, Fankhaenel T, Bojanowski S, Bergmann A. Gaining information about home visits in primary care: Methodological issues from a feasibility study. BMC Fam Pract. 2014;15(1). 10.1186/1471-2296-15-87 . PubMed PMID: 24884460. PMC4018962 24884460

[56] Adams EJ, van Doornewaard A, Ma Y, Ahmed N, Cheng MK, Watz H, et al. Estimating the Health and Economic Impact of Improved Management in Prevalent Chronic Obstructive Pulmonary Disease Populations in England, Germany, Canada, and Japan: A Modelling Study. Int J COPD. 2023;18:2127–46. 10. 2147/COPD.S416988 PubMed PMID: 37789931. 10.2147/COPD.S416988 PMC10543939 37789931

[57] Brecher Dominika Urbanski-Rini Volker Amelung Projektleitung Volker ALE. Rhön Stiftung Studie Hospital@Home [Internet]. [cited 2026 Jan 13]. Available from: https://idw-online.de/de/attachmentdata111357.pdf

[58] Gehrmann J, Hahn F, Stephan J, Steger A, Rattka M, Rudolph I et al. Current Use, Challenges, Barriers, and Chances of Telemedicine in the Ambulatory Sector in Germany-A Survey Study Among Practicing Cardiologists, Internists, and General Practitioners. 10.1089/tmj.2024.0528 39909467

[59] Howcroft M, Walters EH, Wood-Baker R, Walters JA. Action plans with brief patient education for exacerbations in chronic obstructive pulmonary disease. Cochrane Database of Systematic Reviews. John Wiley and Sons Ltd; 2016. 10.1002/14651858.CD005074.pub4 . PubMed PMID: 27990628. PMC6463844 27990628

[60] Patel K, Janssen DJA, Curtis JR. Advance care planing in COPD. Respirology. 2012;17:72–8. 10.1111/j.1440-1843.2011.02087.x 22008225

[61] Macpherson A, Walshe C, Vyas A, O’donnell V. The views of patients with severe chronic obstructive pulmonary disease on advance care planning: A qualitative study. Palliat Med. 2013;27(3):265–72. doi:10.1177/0269216312440606 PubMed PMID: 22450158. 10.1177/0269216312440606 22450158

[62] Yohannes AM, Alexopoulos GS. Depression and anxiety in patients with COPD. Eur Respiratory Rev Eur Respiratory Soc. 2014;345–9. 10.1183/09059180.00007813 . PubMed PMID: 25176970. PMC4523084 25176970

[63] Jácome C, Figueiredo D, Gabriel R, Cruz J, Marques A. Predicting anxiety and depression among family carers of people with Chronic Obstructive Pulmonary Disease. Int Psychogeriatr. 2014;26(7):1191–9. 10. 1017/S1041610214000337 PubMed PMID: 24621411. 10.1017/S1041610214000337 24621411

[64] Cossette S, Lévesque L. Caregiving tasks as predictors of mental health of wife caregivers of men with chronic obstructive pulmonary disease. Res Nurs Health. 1993;16(4):251–63. 10.1002/nur.4770160404 . PubMed PMID: 8378555. 8378555

[65] Yohannes AM, Casaburi R, Dryden S, Hanania NA. The effectiveness of pulmonary rehabilitation on chronic obstructive pulmonary disease patients with concurrent presence of comorbid depression and anxiety. Respir Med. 2022;197. 10.1016/j.rmed.2022.106850 . PubMed PMID: 35427843. 35427843

[66] Zhang X, Yin C, Tian W, Lu D, Yang X. Effects of cognitive behavioral therapy on anxiety and depression in patients with chronic obstructive pulmonary disease: A meta-analysis and systematic review. Clinical Respiratory Journal. Blackwell Publishing Ltd; 2020. pp. 891–900. 10.1111/crj.13226 PubMed PMID: 32510764. 32510764

[67] Curtis JR, Engelberg RA. Communication About Palliative Care for Patients With Chronic Obstructive Pulmonary Disease [Internet]. Available from: www.thoracic.org/copd 16334970

[68] Halliwell J, Mulcahy P, Buetow S, Bray Y, Coster G, Osman LM. GP discussion of prognosis with patients with severe chronic obstructive pulmonary disease: a qualitative study. Br J Gen Pract. 2004 54:904-908. PMC1326107 15588534

[69] Curtis JR. Palliative and end-of-life care for patients with severe COPD. Eur Respir J. 2007; 32(3);796–803. 10. 1183/09031936.00126107 PubMed PMID: 17989116. 10.1183/09031936.00126107 17989116

[70] Tavares N, Jarrett N, Hunt K, Wilkinson T. Palliative and end-of-life care conversations in COPD: A systematic literature review. ERJ Open Res Eur Respiratory Soc. 2017. 10.1183/23120541.00068-2016. PMC5407435 28462236

[71] Tripodoro Vilma A, Bastos F, GE, RJ. and CC. Organization of Palliative Care in Different Parts of the World. In: MacLeod Roderick Duncan and Van den Block L, editor. Textbook of Palliative Care [Internet]. Cham: Springer Nature Switzerland; 2024. pp. 41–82. Available from: 10.1007/978-3-031-48991-4_6 doi:10.1007/978-3-031-48991-4_6.

