# Supplementary material for: Needs among persons with advanced COPD and their informal caregivers: a multi-perspective qualitative study focusing on the potential role of palliative care
Source: BMC Pulm Med. 2026 Oct 19;26:484. doi: 10.1186/s12890-026-04828-5 (PMC13681904; doi:10.1186/s12890-026-04828-5)
Supplement: Supplementary file 1 [file 12890_2026_4828_MOESM1_ESM.docx]

**Table 3** – Spontaneous thoughts when hearing “palliative care” among persons with advanced COPD and informal caregivers who had not received PC yet.

| **Topic** | **Verbatim quotes (spontaneous associations with the term “PC”)** | | **Participant** |
| --- | --- | --- | --- |
| Approaching end of life  Hopelessness | Q1 | What’s the first word? SIDETRACK. I mean, it just feels so final. | Female patient, 75 years old |
| Approaching end of life | Q2 | Yes, that you’re going to die soon, you know? | Female patient, 63 years old |
| Approaching end of life | Q3 | That’s, well, being on the point of dying, you know? So, briefly / well, it’s just that the time period is very limited, as long as you’re still alive. But that’s it now, right? And now just a little bit more, you know? Yeah. | Female patient, 65 years old |
| Approaching end of life  Previous experience (e.g. with relatives) | Q4 | Yes, then it’s usually already coming to the end, right? (...) Yes, that’s palliative care for me. I saw it with my father-in-law. He received palliative care in the hospital for 14 days, and then he was gone. He had passed away. Yes. | Male patient, 67 years old |
| Lack of knowledge | Q5 | I have no idea what it is. | Female patient, 57 years old |
| Lack of knowledge  End-of-life support | Q6 | Not really. I mean, I’ve heard of it before, but I can’t really picture what it actually involves. […] That is, well, I’ll put it this way. What I’ve always imagined it to be is something like end-of-life support. But I don’t know if that’s actually correct. So… (laughs) | Male patient, 62 years old |
| Lack of knowledge | Q7 | Honestly, nothing at all. (laughs) I can’t make any sense of it. | Male informal caregiver, 27 years old |
| Negative association  Lack of knowledge | Q8 | P: Negative.  KS: Why? What do you imagine it to mean?  P: The word itself already sounds negative. Yes, I don’t know if I’m even correct, but it… yeah, it sounds negative. And honestly, I don’t really know what it actually means. | Male patient, 73 years old |
| End-of-life support  Avoidance | Q9 | Yes, assisted dying, I think so now. Yes, and we didn’t really deal with that at all. I haven’t thought about it yet, not at all, not at all, really not, no. (laughs) | Female informal caregiver, 67 years old |
| Hospital ward  Last option | Q10 | Yes, well, we’ve already looked into that, or rather I have. At the [name 1] hospital, that’s possible. There’s a dedicated ward there, and if there’s really no other option, we could transfer things there.  So that means from [name 2], from the pulmonary ward, it’s only one floor down to the palliative care unit, and that would be the last option if it came to that. Yes, actually, I even know the ward. | Male informal caregiver, 58 years old |
| Approaching end of life  Hopelessness  Cancer  Has positive and negative aspects  Feels insufficiently informed to decide | Q11 | Palliative care? Well, from the beginning, when I heard about it, it felt like the final station, like there’s no hope from one side. But from another side, it’s something positive. It makes all this back-and-forth easier. But behind this word PALLIATIVE, of course, there are a thousand questions: Will he still get all the necessary medical aids like everyone else? Or is it just “the end,” and he will only get calming medication and things like that? That’s why I couldn’t decide. My daughter says, “He doesn’t want it.” And I am somewhere in between yes and no. I can’t decide this today or tomorrow. Because what if…? Then he would never go to a doctor again, which is also bad. For him it means very little. We know, it’s like with cancer patients: they have reached the final station. It’s a mix of something positive, people come to you and make things easier, and something negative, these are only the very sick people who will die soon. Not tomorrow or the day after, of course, but still not a good ending. And that’s why I can’t make this decision *now*. I received a letter. And our doctor said: “He is PV”, or what is it called? [SAPV]. And then I just didn’t feel at peace with it. And he doesn’t want it. For now he says no. Of course, it’s exhausting. […] But this palliative unit or palliative care still brings many question marks for both of us. We have read about it, but we still need to find out: will he get the same chance as in the hospital, for example, getting an infusion at home? And what about his back, or if he gets pneumonia or an infection? They cannot save him here at home. What does that mean? That he would never go to the hospital again? So… is it good or bad? I don’t know. It has both sides, positive and negative. That’s my opinion. | Female informal caregiver, 52 years old |
| When life is limited  Not only for the last days of life  Decision autonomy  Previous experience (e.g. with relatives)  Advanced care planning directives | Q12 | That my husband can no longer walk, that he’s bedridden, and that palliative care can extend over several years. […] I have a contract here that’s been ready for three years. The doctor [name], my husband, and I only need to sign it when the time comes. […] My husband needs to know what palliative care MEANS. Palliative means that the patient / I’ve already done this with my dad. Yes, I provided palliative care for my dad. And I’m doing the same now with my husband. I know that the patient’s wishes must always be considered. I know that my husband can no longer be invasively ventilated. And then you can figure out the rest on your own. You don’t need to plan much beyond that. | Female informal caregiver, 67 years old |
| Avoidance | Q13 | Yes. Yes. That I’m in good hands with my doctor because he also provides palliative care. Period. | Male patient, 71 years old |
| Cancer  Avoidance | Q14 | Yes, cancer, right? Well, what am I supposed to say about that? I can’t really say anything about it, you know? Nobody wants to go there, right? Well. And if you have to, then you just have to deal with it, right? But I don’t even want to think about it. Not something like that on top of everything else, right? What I already have is enough. | Female patient, 78 years old |
| Adding more life to life  Hospice | Q15 | Well, I’m not quite neutral. And that’s really the idea behind palliative care / It is / They also have the palliative care staff and the hospices, and their motto is “adding more life to life.” […] That’s what I would want for myself as well. […] Making the most of life once again, doing what I enjoy, being with the people I want around me, maybe fulfilling one last wish and experiencing that wish fully and consciously. That’s what I associate with palliative care. | Female informal caregiver, 66 years old |

**Abbreviations and acronyms:** PC: Palliative care; COPD: Chronic obstructive pulmonary disease; Q: Quote; SAPV: Specialized outpatient palliative care.
