# Supplementary material for: Needs among persons with advanced COPD and their informal caregivers: a multi-perspective qualitative study focusing on the potential role of palliative care
Source: BMC Pulm Med. 2026 Oct 19;26:484. doi: 10.1186/s12890-026-04828-5 (PMC13681904; doi:10.1186/s12890-026-04828-5)
Supplement: Supplementary file 2 [file 12890_2026_4828_MOESM2_ESM.docx]

**Table 4** – Spontaneous thoughts when hearing “palliative care” among persons with advanced COPD and informal caregivers who were currently receiving or had received PC.

| **Topic** |  | **Verbatim quote** | **Participants** |
| --- | --- | --- | --- |
| End-of-life support  Hospice  Supportive  Supports informal caregivers | Q1 | Exactly, that’s what I looked up and I educated myself about it. And now / what it is? End-of-life support, exactly like a hospice also accompanies the dying process. And it supports the relatives as well. And… well, there was nothing unusual for me in that. Because others see it as something connected with fear, I less so. Right? Because I know exactly what it is. And of course, I’m glad that I can turn to them with any question. That makes me happy again, because I didn’t have that before. Right? I have to say that. I’m glad I have support. | Female patient, 69 years old |
| Approaching end of life  Previous experience (e.g. with relatives)  Hopelessness  Informed by PC team  Good/better care | Q2 | The road to death. Because actually, I didn’t know anything about palliative care before. My sister died four years ago. I visited her once more in the hospital, and on the way to the ward my niece said to me, “They’ve put [name] in palliative care now.”  To me, that meant she was sitting there on her bed, basically helpless. And in my mind, palliative care also means that if I, as a palliative patient, have to go to the hospital, I end up on a special ward where people think, “Oh, just let them lie there, there’s nothing more we can do anyway.”  They all tell me that’s absolutely not the case, that I would receive special support there. That’s what the woman from the palliative team who came here said. Someone came and explained everything to us, the doctor and the medical assistants. They all contradicted me. They said it’s not like that. That I would actually get more help, that they would take better care of me.  But for me, palliative care has always meant the road to death, yes. | Male patient, 67 years old |
| Alleviation of pain/suffering/relief  Advance care planning directives | Q3 | That the path to a RELATIVELY easy death is opened up for me. You see, I had to, because I was in terrible pain, and then / I had to call emergency services. Or rather, my son called them. They found out it was gallstones. And then I / four weeks later or so, I had to have surgery. As a PRECAUTION against the pain that might come, which I didn’t want / didn’t want to go through again, I made an advance directive stating that if I didn’t wake up from the anaesthesia, no measures should be taken to bring me back or keep me ALIVE. That’s written everywhere in my directive. No life-sustaining or life-prolonging measures. All the doctors have received this information and are aware of it. That means, for example, if I ever have to undergo another gallbladder operation. But it was because of the threat of that pain. Like I said, I really wouldn’t want to go through that again. | Male patient, 80 years old |
| End-of-life support  Approaching end of life  Alleviation of pain/suffering/relief | Q4 | Yes, we’ve already done and arranged all of that. He was connected to the palliative care network, but in the meantime he was discharged from it. Basically because they said, “In the end, it’s not really at the point where he would even need to start thinking about receiving palliative care yet,” right?  So, as I said, he was connected to the palliative network. Otherwise, of course, at some point it will come to that. And that’s how it should be. And then you support him through it.  But as I said, I’m aware of what palliative medicine means, what it is, and that it WILL become necessary at some point. And it’s absolutely important and right that he’s connected to it, because for him there’s really nothing worse than the fear of suffocating to death. So it’s clear that eventually the time will come, right?  But for me, it honestly still feels like it’s a long way off, because in terms of his condition, even if he personally feels worse, I actually think objectively he’s still far enough away from that point. | Male informal caregiver, 43 years old |
| Approaching end of life  Alleviation of pain/suffering/relief | Q5 | Before, my first thought was death. Nowadays, my first thought is relief, so life before and life after, you know? | Female informal caregiver, 37 years old |
| Alleviation of pain/suffering/relief  Informed by PC team  Advance care planning directives  Autonomy | Q6 | No, I wouldn’t know what. The patient has to cooperate. It’s not just up to the doctors or the palliative care staff. If I were stubborn… I still smoke, I admit it. But they said that’s okay, at least you’re honest about it. […] And then they said, “Well, that’s just how it is. But you still have the right to live. And above all, to live reasonably well. You don’t have to suffer.” […] And then there’s still the option that they can put me to sleep, they explained to me what that means, kind of like a coma. And that’s something I can accept. I think that’s a good idea. But that’s an option that was offered to me. I could choose it. The palliative care team or a doctor doesn’t decide that. […] I’m the one in control. I can decide that myself. And that really means something to me, that nothing will be done against my will. | Male patient, 57 years old |

**Abbreviations and acronyms:** PC: Palliative care; COPD: Chronic obstructive pulmonary disease; Q: Quote; SAPV: Specialized out-patient palliative care.
