# Supplementary material for: Needs among persons with advanced COPD and their informal caregivers: a multi-perspective qualitative study focusing on the potential role of palliative care
Source: BMC Pulm Med. 2026 Oct 19;26:484. doi: 10.1186/s12890-026-04828-5 (PMC13681904; doi:10.1186/s12890-026-04828-5)
Supplement: Supplementary file 3 [file 12890_2026_4828_MOESM3_ESM.docx]

**Table 5 -**  Barriers to access palliative care (PC) for persons with advanced chronic obstructive pulmonary disease (COPD) and their informal caregivers, verbatim quotes from formal caregivers, listed according to categories of the coding scheme.

| **Topic** |  | **Verbatim quotes** | **Participant** |
| --- | --- | --- | --- |
| Misunderstandings or lack of knowledge about PC itself or its available structures among physicians | Q1 | Well, I suspect that the need is actually significantly greater than what we see. After all, I really only see the COPD patients who have actually found their way to us. But because / It is such a common chronic illness that there are certainly many who know nothing about it. And that is also due to the fact that, I believe, perhaps many general practitioners, who could establish this initial contact, are sometimes not even aware that COPD is now indeed treated within a palliative context, right? They may still, in a way, follow the old school and initially refer only oncological patients. And I think that of course leads to us having many COPD patients at home who are not seen at all. But I can only speculate about that, right, because, as I said, they (laughs) do not find their way to us. I do not know them at all, right? | Female PC-physician, 13 years of experience |
| Misunderstandings or lack of knowledge about PC itself or its available structures among physicians  Insecurities regarding the adequate time to initiate PC for people with advanced COPD | Q2 | Well, in palliative care, I understand it less in terms of COPD or respiratory diseases; rather, I see it as a non-curative, symptom-relieving therapy for patients with very severe conditions. This also includes cancer patients or patients in severe pain. Of course, you have to say that COPD in an advanced stage is an incurable disease that can only be approached palliatively. That’s true. But I think the term ‘palliative measures’ isn’t something we actually use very often. Or maybe we don’t really take it into account in our daily routine. But that’s certainly a point that could be defined more precisely and perhaps discussed again, what exactly is meant by palliation in a patient with advanced COPD. | Male pulmonologist, 31 years of experience |
| Misunderstandings or lack of knowledge about PC itself or its available structures among physicians | Q3 | Well, in very advanced stages of the disease, especially when patients are already severely functionally impaired. So, severely reduced lung function, older age, comorbidities, oxygen therapy. And when they simply can’t cope well anymore and are functionally so limited, then early palliative involvement would definitely make sense. But that’s done very rarely. Most pulmonologists, especially those in private practice, have no training in palliative medicine at all. Most general practitioners are the ones who mainly have that training. Fewer specialists provide this kind of care, like SAPV, and they often don’t have additional training in palliative medicine either. So early palliative involvement in advanced disease would make sense for symptom relief. It’s not that we’re saying they’re about to die or that they have to go to a hospice or a palliative care unit. But palliative medicine here is still strongly associated with oncological diseases, that it’s all about cancer. Yet in pulmonology there are many conditions where early palliative therapy would also be beneficial, such as COPD or pulmonary fibrosis, for example. These are also chronic, progressive diseases that eventually lead to death. And early palliative co-management would certainly be helpful. | Male pulmonologist, 31 years of experience |
| Misunderstandings or lack of knowledge about PC itself or its available structures among physicians  Misunderstandings about, lack of interest on or rejection of the topic PC and/or lethality of COPD among patients | Q4 | So I think it is simply also a lack of information [among physicians]. […] I believe it is perhaps also a lack of information among patients. And then, of course, sometimes it is really also a matter of personality, that this word, the term palliative medicine, is still perhaps negatively associated among the general population and accordingly among patients, and really seen only as end-of-life medicine, and therefore the patient does not even want or cannot bear to hear it. Or the relatives, right? | Female PC-physician, 13 years of experience |
| Misunderstandings or lack of knowledge about PC itself or its available structures among physicians  Misunderstandings about, lack of interest on or rejection of the topic PC and/or lethality of COPD among patients | Q5 | I’ve prescribed it [SAPV] very often for oncology patients, but they often also have COPD. However, for COPD patients, I’d say it’s prescribed rather rarely. […] Because it’s not really present in people’s minds that it’s a fatal disease that ends life. (…) That’s how it is for patients and also in society in general. | Female pulmonologist, 12 years of experience |
| Misunderstandings or lack of knowledge about PC itself or its available structures among physicians  Misunderstandings about, lack of interest on or rejection of the topic PC and/or lethality of COPD among patients | Q6 | I think, first and foremost, that it is not very well known, that is, that it is not yet, in quotation marks, a “popular” topic like it is now with oncological diseases. Palliative care is already more common there than with the chronic disease COPD. Perhaps it is also not so well known that this disease can potentially end fatally. | Female GP, 9 years of experience |
| Misunderstandings or lack of knowledge about PC itself or its available structures among physicians | Q7 | Barriers? Well, barriers, there aren’t any major barriers. As I said, I’m not familiar with such palliative care facilities. So if you say, ‘That’s nonsense, you have no idea, we have five options here in [city],’ then I’m simply uninformed, but my colleagues are too. But basically, the real barrier is that there are very few options available. And if there are options, there are certainly possibilities to prescribe breathing exercises or pulmonary rehabilitation sports. And for us, there really isn’t any barrier to that, because we definitely do it. So I don’t see a barrier to the process itself from my position, but rather a lack of available services. | Male pulmonologist, 31 years of experience |
| Uncertainty regarding the adequate time to initiate PC for people with advanced COPD | Q8 | When I signed up for this, I thought about it, because I had actually never enrolled a patient palliatively just for COPD. Usually, it was only when they had / or when a tumour disease was present, or when the shortness of breath couldn’t be controlled. When they basically always / when they needed a phone number so someone could come by and inject morphine and so on. That’s the point for me when I enrol them. Probably actually too late then, right? (…) […] I have to say that the others [patients] have been STABLE for four years, simply, with the oxygen device. And you can’t enrol them palliatively for that long, right? (…) So for ME, I think the time comes when I feel that I can’t manage it / they aren’t well-handled if they only have me. That’s it. So that’s the point for me, when they say: “WOW, the weekend was terrible. And I considered calling an ambulance. But actually, I don’t want to go to the hospital anymore.” That would be the moment when I say: “Yes, how about palliative care? There’s something available, right?” Yes. Actually maybe this conversation first with the relatives, because they might be afraid: “What do I do if they suddenly have shortness of breath?” Then I say: “Yes, you have to see. If it’s acute right now, then THIS way. But fundamentally, there’s also the option that…” because it is an incurable disease. I talk about that with them quite often. And then, when they say: “Yes, that would be something,” I bring it up in the next conversation with the patient, that this option exists. But for just COPD, actually it hadn’t really occurred to me yet. Yes. | Female GP, 14 years of experience |
| Uncertainty regarding the adequate time to initiate PC for people with advanced COPD | Q9 | That people don’t think about the fact that patients repeatedly go through the “revolving door” of hospital, exacerbation, back again, so that the right moment, when palliative care is indicated, is missed, and that some patients may also not be ready. That is sometimes difficult. If you address it early, then perhaps / it is often clearer for the patients that in a certain situation they no longer want to go to the hospital as an emergency, but would rather follow the palliative path. When the right time has come is very individual; it has to be discussed. Usually, after two or three exacerbations with hospital stays and all the fuss, and each time coming out worse than they went in, the patient realizes: What do I still want from that? But it doesn’t have to get that far. So, for one person earlier, for another later. It is difficult / it is simply difficult to find the right moment. | Male GP, 28 years of experience |
| Uncertainty regarding the adequate time to initiate PC for people with advanced COPD | Q10 | We have the experience that it is problematic to establish a life expectancy prognosis in many COPD patients. Therefore, the treatment of these patients or their admission into a palliative medical system is difficult. […]  Yes, the difficulty lies precisely in the fact that even in patients with severe COPD we cannot estimate life expectancy well, that patients often still have a will to live and a desire for therapy and therefore frequently wish to be admitted to hospital in crises. And we only care for patients at home, so we can only help to a certain extent, but in a crisis the patients then do go to the hospital. | Male PC-physician, 28 years of experience |
| Uncertainty regarding the adequate time to initiate PC for people with advanced COPD | Q11 | Well, I mean, I work here in the pulmonary specialty clinic, so overall we have very many patients, including those with advanced COPD. Therefore, of course, we have a lot of experience. It is, in principle, I would say, also difficult, especially because / as this also relates to the palliative situation or severe COPD, simply to determine WHEN this point comes, that is, when one somehow moves in the direction of palliative medicine for COPD patients. So, it is significantly easier, for example, with patients with advanced lung cancer than, for example, with patients with COPD, to decide when exactly to set this boundary, yes, and then somehow also change the therapy or have conversations with the patients in this direction. | Female pulmonologist, 8 years of experience |
| Uncertainty regarding the adequate time to initiate PC for people with advanced COPD | Q12 | These people live too long. It is not easy to determine when the right time for palliative care is. Sometimes it is too early, sometimes too late. Some young people live for a very long time with such COPD, for example. They manage for a very long time because the nursing situation at home is optimal. The care is optimal. Sometimes, however, palliative care is initiated much too late, namely after a stay in an intensive care unit and shortly after intubation. And then it is said that the person is going home to die. Could you please step in now. So, of course, that is late and you do not know the person. That involves severe suffering. So, the timing of the start of palliative co-care is difficult. | Female PC-physician, 27 years of experience |
| Uncertainty regarding the adequate time to initiate PC for people with advanced COPD  Unsatisfactory case management and communication | Q13 | So I think, pulmonologists, in my experience, are not that open about the prognosis, because in stage 4 it is quite poor. So five-year survival rate, I don’t know, a few percent in any case. And also the one-year survival rate is not good. And that this is stated so clearly by non-palliative care physicians is not known to me. And even for palliative care physicians it is actually sometimes difficult to assess. One / so one usually won’t name an exact time period anyway, but that is difficult. So that it is a life-limiting illness is, I think, rather rarely addressed by pulmonologists, more often by palliative care physicians. And from general practitioners, I honestly don’t know. | Female GP, 19 years of experience |
| Unsatisfactory case management and communication | Q14 | Oh, the barriers. Yes, perhaps interdisciplinary ones. Sometimes you don’t really have direct access yourself. Within the framework of the DMP, a lot also runs through the staff, they have their own access to the patients, as do physiotherapists or other specialists. Well, patients who are still mobile are sometimes also under specialist care. And everyone has to look at their own barriers and how to deal with them.  On the other hand, in the end, if barriers are there, then they’re just there. It’s about dealing honestly with the facts and what those facts mean for the patient, how things might progress. I think you have to be honest with yourself and focus on how to achieve good symptom control. You have to look at what actually helps.  With COPD, in advanced stages, things are often already relevant that you would normally only see in palliative patients: giving morphine, ventilators, physiotherapy, breathing therapy, all of that. So everything that might only come into play for other patient groups in a palliative situation has to be addressed much earlier in treatment here. | Male GP, 28 years of experience |
| Unsatisfactory case management and communication  Misunderstandings about, lack of interest on or rejection of the topic PC and/or lethality of COPD among patients | Q15 | The multisegmented management. So that, in practice, you bring in all those involved into the case and coordinate it. […] Often the patient doesn’t want to take up the services at all, due to the depression, with the social withdrawal. Then there are often time constraints within the family environment or regarding caregiving. And then it is also incredibly difficult to get the individual actors, the players involved in the healthcare system, around one table. It often ends up resting solely on me as the general practitioner. And yes, it is difficult to motivate these patients, because they often also have a severe accompanying depression. | Male GP, 24 years of experience |
| Misunderstandings about, lack of interest on or rejection of the topic PC and/or lethality of COPD among patients | Q16 | Even if you have very honest and open conversations with these patients, or with the relatives, it is still / Interestingly, the support through SAPV is EASIER for oncological patients. Because apparently this is still much more socially accepted. When a person has cancer, then in people’s minds it somehow more readily moves in the direction of dying at some point. That is simply not the case with COPD. And accordingly, these patients are somewhat more difficult to engage in such care, or the relatives as well. | Female pulmonologist, 34 years of experience |
| Misunderstandings about, lack of interest on or rejection of the topic PC and/or lethality of COPD among patients | Q17 | Yes, they are somewhat, how should I put it, in part, not all, in part rather, yes, one says indolent, so not very perceptive regarding their own symptoms. And also, what do I know, offers to meet more frequently and perhaps also discuss a palliative application are not taken up. But I already hinted at it just now, of course that cannot be applied to everyone. There are also exceptions, where you then notice that they come in time. But in my perception that is rather the / the smaller proportion. | Female GP, 9 years of experience |
| Misunderstandings about, lack of interest on or rejection of the topic PC and/or lethality of COPD among patients | Q18 | Yes, sometimes also / a limited understanding of the illness or, yes, also a bit of denial mechanisms, not seeing the severity of the illness. Because it is not a cancer, and you have been living with it for decades and it probably won’t be that bad. | Female GP, 19 years of experience |
| Misunderstandings about, lack of interest on or rejection of the topic PC and/or lethality of COPD among patients | Q19 | So, some are unfortunately still young. But they cannot imagine that it is coming to an end or could come to an end. Therefore, with every exacerbation they want to go to the hospital. And no matter what support is available at home, the emergency doctor is called and the person is intubated after all, for example, because they cannot let go. That is limited insight into the illness. Yes, if there is acceptance of the severity of the disease, a palliative concept is still sometimes difficult. | Female PC-physician, 27 years of experience |
| Misunderstandings about, lack of interest on or rejection of the topic PC and/or lethality of COPD among patients | Q20 | [With] cancer patients, that works somehow at some point. People know that at some point, that / cancer patients eventually go to the hospice. But for COPD, they have / So, nobody wants to hear that, right? So, nobody wants to hear: This is the end now. | Female GP, 30 years of experience |
| Misunderstandings about, lack of interest on or rejection of the topic PC and/or lethality of COPD among patients | Q21 | Because many patients are simply AFRAID when you say it / right, when you say: “palliative care.” And then many of them think: “Oh, now I’m already heading toward dying.” But that is often not the case at all, and that’s not what is meant. That means it also takes time to explain it, right? What is meant by it and what the benefit for the patients is. And that it is a good thing. | Female pulmonologist, 13 years of experience |
| Lack of time to discuss PC with patients and informal caregivers | Q22 | I think the biggest barrier is simply TIME. For me, a COPD patient is always very time-consuming once they are in the room. It often takes a bit longer because they might not be able to walk well, need more time to get to the consultation room, so I think I often just need more time. And that would already be the answer to the solution. In other words, you have to take a bit more time. Normally, we schedule 15-minute appointments for standard consultations. And if a patient with shortness of breath comes in and you think about admitting them, you often don’t have, how should I say, a cooperative patient. And to explain things thoroughly, or to grasp the complexity of the disease, I sometimes really need more time, and in my view, that would already solve the problem. | Female GP, 9 years of experience |
| Lack of time to discuss PC with patients and informal caregivers | Q23 | So, we have between three and five minutes per patient. And during that time, a lung function test might even have to be done. That means we / of course, there are individual cases where you take more time when necessary, and we do that. But generally, it’s really not the case that we, or my staff, have enough time to provide patient education, make preventive recommendations, conduct training sessions, review medications / if we were to do all of that very regularly and thoroughly, it would require a whole system and staffing structure that we simply cannot provide.  So we do try to do it properly. And I would say we succeed to some extent. But there’s a significant lack of time and also of resources. | Male pulmonologist, 31 years of experience |
| Lack of time to discuss PC with patients and informal caregivers | Q24 | Yes, well, no / there’s definitely not enough time available, that’s absolutely clear. Ideally, you’d need at least 20 to 30 minutes for something like an initial counselling, right? So you can really get to know the patient, understand what has already been done and what hasn’t, consider which options are appropriate, and provide proper information and counselling, yes. | Female pulmonologist, 13 years of experience |

**Abbreviations and acronyms:** PC: Palliative care; COPD: Chronic obstructive pulmonary disease; Q: Quote; GP: General practitioner; SAPV: Specialized outpatient palliative care.
