# Supplementary material for: Needs among persons with advanced COPD and their informal caregivers: a multi-perspective qualitative study focusing on the potential role of palliative care
Source: BMC Pulm Med. 2026 Oct 19;26:484. doi: 10.1186/s12890-026-04828-5 (PMC13681904; doi:10.1186/s12890-026-04828-5)
Supplement: Supplementary file 4 [file 12890_2026_4828_MOESM4_ESM.docx]

**Table 6** – Suggestions to improve access to PC for persons with advanced COPD and their informal caregivers, verbatim quotes from formal caregivers, listed according to categories of the coding scheme.

| **Topic** | **Quote** | **Verbatim quotes** | **Participant** |
| --- | --- | --- | --- |
| Education and informational services and resources | Q1 | So, in general, it should be such that already in medical school, well, that is now also established, that one already learns in school what palliative care is and who belongs to it and how it can be conveyed and who to turn to. And then it should also be, if / The patients come incredibly often to the hospital repeatedly with re-admission and then discharge and re-admission, that it basically / If it is once stated in the discharge report that they are stage 4, that they are then automatically, so to speak, presented to a palliative care physician in the hospital as well, so that he / so that he can properly manage the patients. Because it is also about ensuring that they are well managed with their shortness of breath with medications, things like that, right, and so that there is simply attention given to the fact that this exists. And that it does not simply mean that you will soon die, but that you are simply well cared for, so to speak. | Female pulmonologist, 8 years of experience |
| Education and informational services and resources | Q2 | I also offer training for the emergency teams every two years. And it is called, I think, last aid or first aid, last aid or something similar. In any case, that the teams also know about seriously ill people, because they are often called for such patients as well. | Female PC-physician, 27 years of experience |
| Education and informational services and resources | Q3 | So, in general, it should be such that already in medical school, well, that is now also established, that one already learns in school what palliative care is and who belongs to it and how it can be conveyed and who to turn to. | Female GP, 19 years of experience |
| Education and informational services and resources | Q4 | This word, the term palliative care is also still maybe negatively perceived in the general population and therefore by the patient, and really only seen as this dying medicine, and that’s why the patient doesn’t even want to hear it and can’t. Or the relatives, right? That means that not only the / on the medical side needs to be informed, but also simply the knowledge about palliative care in the general population, what it means, or what it MEANS TODAY / That it is not only about dying, (laughs) but also about good symptom control, about as much time at home as possible. That simply also needs to become more public or be more integrated into general knowledge, so that patients can hear it and accept it. | Female PC-physician, 13 years of experience |
| Education and informational services and resources | Q5 | We can explain that it is not only the end of life, but an extension of measures. […] It is important to stay in close contact with the patients, to explain more, to give flyers, to provide information, so that they understand what palliative care physicians bring for patients. So then they can make a good decision. | Male pulmonologist, 15 years of experience |
| Education and informational services and resources | Q6 | But perhaps a counselling hotline, just off the top of my head, whether through self-help organizations, health insurance providers, or patient associations. Or that maybe one gives those affected and their families the possibility to call someone and say: “Listen, I have this issue here. Many others probably have it too. What are your experiences with it? Can you give me some advice?” So as not to have the feeling of standing there alone, right, and having to go through this limitation as the FIRST person in the world. Because that is definitely not the case. But I have the impression that many feel that way. They seem very isolated. There is little exchange happening, at least that I would be aware of, with other affected people, with other affected families, right? That would be something that could perhaps also be better networked, right? Or also an app, yes, maybe that would also be a possibility, a health app with counselling, with chat function, right? I could well imagine that this could also be a great help. | NP26_pneumo |
| Education and informational services and resources | Q7 | Yes, preferably the health insurance, but since they’re already stingy with money anyway, they might not be the best contact after all. Yes, I don’t even know if there’s anything else / I know there’s independent care counselling. Here in our city, Caritas [a charitable social-service organization] definitely does that. But I think they have waiting times of about three or four weeks. So yes, maybe one could / so expanding care counselling would certainly be a good thing. | Female GP, 9 years of experience |
| Better case management | Q8 | And then it should also be, if / The patients come incredibly often to the hospital repeatedly with re-admission and then discharge and re-admission, that it basically / If it is once stated in the discharge report that they are stage 4, that they are then automatically, so to speak, presented to a palliative care physician in the hospital as well, so that he / so that he can properly manage the patients. Because it is also about ensuring that they are well managed with their shortness of breath with medications, things like that, right, and so that there is simply attention given to the fact that this exists. And that it does not simply mean that you will soon die, but that you are simply well cared for, so to speak. | Female GP, 19 years of experience |
| Better case management | Q9 | So, for example from the hospital, that they also / are also advised by a social worker and that one speaks about it EARLY on, that there is support from palliative care / from the palliative care side. And that one involves it, and, yes, simply through a lot of talking. | Female pulmonologist, 12 years of experience |
| Better case management | Q10 | I believe the majority of people actually need a referral from general practitioners or also from office-based pulmonologists or even from acute-care hospitals. So, that’s what I would wish for there. And I think that, again, it only works through communication, that we have the opportunity to make palliative care services truly known to other physicians in a completely VALUE-FREE way. In order to also be able to convey the information: what can medicine actually mean in terms of improvement or relief for these patients? | Male PC-physician, 21 years of experience |
| Better case management | Q11 | What I find good is this surprise question, which is also used for tumor patients. […] And, if the answer is no, and this person has symptoms, is suffering. And unfortunately COPD patients do [suffer] at the end of life, then this person would benefit from palliative counseling, even co-care. And for me there is also nothing against it if they continue to have their non-invasive ventilation in parallel or can still go to the pulmonologist. So that can also take place in parallel. | Female PC-physician, 27 years of experience |
| Better case management | Q12 | So, one could perhaps include it in the DMP, that you actively address it and have to tick off that you have talked about prognosis and the course of the disease. That would maybe be such a reminder. So, I think a simple reminder aid would really help me in everyday practice, as silly as that sounds. And YES, I think that’s already it. So, I would somehow need a reminder that keeps prompting me to also talk with the patient about where things are actually heading in the long term. | Female GP, 14 years of experience |
| Better case management | Q13 | I think it’s central in such COMPLEX disease situations that you also respond in a complex way, right? So, what about a COPD board? I’ll just call it that. Of course, that also means additional time and personnel effort. That’s an idea where I’d say, I actually find that sensible, right? And as I said, first of all, figuring out what people need. Now maybe not just palliative care-wise, but overall. And how do you better network these needs? I have the impression that currently many people are working very dedicatedly, but in parallel, side by side. And it’s significantly more difficult to achieve networking, so that knowledge gets transferred from one line of treatment to the next. And at the same time, giving the affected people the feeling that it still stays in one hand and has an overall concept, right? | Male PC-physician, 21 years of experience |

**Abbreviations and acronyms:** PC: Palliative care; COPD: Chronic obstructive pulmonary disease; Q: Quote; GP: General practitioner; DMP: Disease management programme.
