# Supplementary material for: Needs among persons with advanced COPD and their informal caregivers: a multi-perspective qualitative study focusing on the potential role of palliative care
Source: BMC Pulm Med. 2026 Oct 19;26:484. doi: 10.1186/s12890-026-04828-5 (PMC13681904; doi:10.1186/s12890-026-04828-5)
Supplement: Supplementary file 5 [file 12890_2026_4828_MOESM5_ESM.docx]

**“Care Needs in advanced COPD: The perspective of patients, informal, and formal caregivers”**

Interview guide for persons with advanced COPD, version 07 (as of 21.02.2024)

**______________________________________________________________________________**

| **Thematic blocks** | **Interview questions** |
| --- | --- |
| **Opening question:**  **Experience of the disease in daily life** | How do you experience your COPD in your daily life?   - *What symptoms do you have as a result of COPD? How have these symptoms changed over time?* - *How has your life changed due to COPD, and how did you first notice these changes?* - *What limitations do you experience in daily life because of COPD? (physical, social, psychological, financial, independence, mobility, etc.)* - *What do you have to do to manage your illness in everyday life?* - *What emotions does living with COPD evoke in you?* |
| **Medical needs**  Evaluation  Needs | When you think about how you experience your COPD in daily life, how satisfied are you with your medical care?   - *How do you experience the communication between yourself and your doctors?*   - *How could this communication be improved or made even better?* - *Are there any questions or topics regarding your COPD and its impact on your life that you would like to discuss with your doctor or other healthcare professionals?*   - *If so, which ones?* - *Do you feel that you are in good medical care? Why or why not?* - *Do you have specific wishes for your doctors or other healthcare professionals? What could they do better or differently?* - *What else would you like in terms of medical support? What do you feel is missing?*   - *Who could fulfil this wish and how? Is there an optimal time for this?* |
| **Psychological / spiritual / social needs**  In everyday life  With regard to the course of disease  [If limitations and/or negative changes were mentioned that have not yet been addressed here] | You told me that COPD triggers (mention feelings) for you in daily life. Are there any other emotions that are triggered in connection with COPD? (e.g., fear [of suffocating], sadness, anger, etc.)  How do you cope with these emotions?   - *What helps you to manage these emotions?* - *What would you wish for to cope with these emotions more effectively?* - *Who could support you in this, and in what way?*   Do you have any concerns or worries regarding the progression of your illness?   - *If so, what are they?*   What helps you, or what support do you currently receive, to cope better with the course of the disease?   - *What type of support (e.g., informational, organizational, psychological, spiritual) would you like to receive in order to cope better with the course of advanced COPD?*   - *Who could provide this support, and in what way?*   You have already told me about your limitations/changes in your life. Are you experiencing any further limitations or negative changes (e.g., regarding relationships, your independence, daily life) due to your advanced COPD?   - *Is there anything that has helped you cope better with these limitations/changes?*   What type of support (e.g., informational, organizational, psychological, spiritual) would you like to receive in order to cope better with these limitations/changes?   - Who could provide this support, and in what way? |
| **[If exacerbations were mentioned]** | You have already told me about your experience with exacerbations. Is there anything that has helped you be better prepared for exacerbations?   - *How do your informal caregivers respond to exacerbations?* - *What information would help you or your informal caregivers cope better with exacerbations?*   - *Who could provide this information?* - *How do your informal caregivers respond to exacerbations?* - *What information would help you or your informal caregivers cope better with exacerbations?*   - *Who could provide this information?* - *What type of support would help you or your informal caregivers cope better with exacerbations (e.g., medical, psychological, organizational, other)?*   - *Who could provide this support, and in what form?* |
| **Organisational / social needs**  Needs of informal caregivers if informal care is being received | You have told me about your experience of living with your COPD in daily life. To what extent do you rely on practical help in daily activities due to your advanced COPD, for example with household tasks, shopping, dressing, dealing with authorities, or attending medical appointments?   - *Who helps you, and with what?* - *To what extent would you like to receive support?*   - *From whom? In what context or form?*   To what extent, in your view, might informal caregivers need information in order to support you better?   - *What kind of information? From whom? In what context or form?*   To what extent, in your view, might informal caregivers need support?   - *What kind of support? From whom? In what context or form?*   *In our study, we are interested not only in the perspective of people with advanced COPD, but also in the perspective of those close to you who support you. Do you think that the person(s) you mentioned would also be willing to participate in a telephone interview as part of our study?*  *[If yes: remind them at the end after handing over the recruitment materials to informal caregivers.]* |
| **Palliative care** [for patients recruited by general practitioners and pulmonologists] | What is your immediate thought when you hear the word “palliative care”?  Palliative care is a form of medical treatment aimed at relieving symptoms and improving the quality of life of patients, enabling a better life despite serious illness. This includes attempts to control symptoms such as pain, shortness of breath, anxiety, or psychological suffering. Do you have any experience with palliative care?   - *If yes, when and how did you learn about it?*   - *Are you currently receiving palliative care for COPD?*   - *How helpful is the palliative care for you?* - *If not, do you think it would help you?*   - *If yes, in what way exactly?* - *To what extent should your informal caregivers be involved in palliative care, for example in discussions or decision-making?* - *At what point do you think palliative care becomes helpful for someone with COPD?* - *Do you have any concerns regarding palliative care?*   - *If yes, what are they?* - *Would you like to receive information about palliative care in general?* |
| **Palliative care**  [for patients recruited by palliative care physicians] | You are currently receiving palliative care. When, how, and from whom did you learn about palliative care?  How helpful is the palliative care for you?   - *Did you have any specific concerns or worries when deciding to receive palliative care?*   In your opinion, at what point would palliative care have been helpful for you?  What role, in your view, should your informal caregivers play in your palliative care?  Do you have any wishes regarding your palliative care for COPD? / Is there anything that could be done differently or better? |
| **Other needs** | Looking back: what would you have wished for when you first learned about your COPD diagnosis? (e.g., more information about the disease, points of contact, additional support services for patients/informal caregivers/supporters)  Are there any further needs you have regarding COPD that we have not yet discussed? |

| Your gender? | 🔾 Male  🔾 Female  🔾 Diverse  🔾 [Does not know]  🔾 [No response] |
| --- | --- |
| In which year were you born? | ____________  🔾 [Does not know]  🔾 [No response] |
| Country of birth | 🔾 Germany (current boundaries)  🔾 Another country:_____________________  🔾 [Does not know]  🔾 [No response] |
| Country of birth of your parents (multiple answers possible) | 🔾 Germany (current boundaries)  🔾 Another country:_____________________  🔾 [Does not know]  🔾 [No response] |
| Marital status:  *“Married,” “Divorced,” and “Widowed” also apply to registered same-sex partnerships. “Separated” means legally separated, not just living apart.* | 🔾 Married / in a partnership  🔾 Married, separated from spouse/partner  🔾 Single  🔾 Divorced  🔾 Widowed  🔾 [Does not know]  🔾 [No response] |
| Are you still being treated for COPD by [name of the recruiting physician]? | 🔾 Yes, _______________________________  🔾 No  🔾 [Does not know]  🔾 [No response] |
| Are you receiving treatment for COPD from anyone else? | 🔾 Yes  🔾 No  🔾 [Does not know]  🔾 [No response]  If yes, could you specify the medical specialties of these providers?  _____________________________ |
| Do you have a care level (Pflegegrad)? | 🔾 Yes: _______  🔾 No |
| Where do you live? | 🔾 In a large city (> 100,000 inhabitants)  🔾 In a small city (20,000 – 100,000 inhabitants)  🔾 In a rural area (< 20,000 inhabitants) |
| *Highest* educational degree | 🔾 Student, attending a full-time general education school  🔾 Left school without completing secondary education  🔾 Secondary school leaving certificate (Hauptschule)  🔾 Intermediate school leaving certificate (Realschule / Mittlere Reife)  🔾 Polytechnic secondary school of the former GDR (8th–10th grade)  🔾 Technical college entrance qualification (Fachhochschulreife)  🔾 General or subject-specific higher education entrance qualification (Abitur / Gymnasium / EOS, including EOS with vocational training)  🔾 Other school degree  🔾 [Does not know]  🔾 [No response] |
| Were you employed? | 🔾 Yes, until the year __________  🔾 No  🔾 I am currently employed  🔾 Full-time employed  🔾 Part-time employed  🔾 [Does not know]  🔾 [No response] |
| EQ-5D-VAS  Now I would like to ask you to tell me how good or bad your health is TODAY.  Please imagine a vertical line with numbers from 0 to 100.  The best health you can imagine is marked with the number 100 at the top of the line.  The worst health you can imagine is marked with the number 0 at the bottom of the line. I would now like you to tell me where on the line you would place your health TODAY.  *(Note for the interviewer: Mark the line at the point that represents the respondent’s health today. Then enter the number corresponding to the marked point in the box below.)*  Respondent’s health today = |  |
| *Clinical COPD Questionnaire (CCQ)*  *Please tick the answer that best describes how you have been feeling over the past 7 days (only one answer per question).*  *How often, on average, over the past 7 days were you:*  1. short of breath when you were **at rest?**  2. short of breath when you have been physically active?  3. **worried** that you might catch a cold or that your breathing problems could get worse?  4. **feeling down** (depressed) because of your breathing problems?  **How often:** 5. have you **coughed** over the past 7 days?  6. Have you had **mucus** in your airways over the past 7 days?  Over the past 7 days, how much were you, on average, limited in the following activities because of your breathing problems?  7. **Strenuous physical activities** (e.g., climbing stairs, hurrying, exercising, carrying heavy loads)?  **8. Moderately strenuous physical activities** (e.g., walking, doing household chores, carrying light loads)?  9. **Everyday activities at home** (e.g., getting dressed, washing, bathing, or showering)?  10. **Social contacts and activities with other people** (e.g., talking, spending time with children, visiting friends/relatives)? | 🔾 Not at all (0)  🔾 Very rarely (1)  🔾 Rarely (2)  🔾 Sometimes (3)  🔾 Often (4)  🔾 Very often (5)  🔾 Almost always (6)  🔾 Not at all (0)  🔾 Very rarely (1)  🔾 Rarely (2)  🔾 Sometimes (3)  🔾 Often (4)  🔾 Very often (5)  🔾 Almost always (6)  🔾 Not at all (0)  🔾 Very rarely (1)  🔾 Rarely (2)  🔾 Sometimes (3)  🔾 Often (4)  🔾 Very often (5)  🔾 Almost always (6)  🔾 Not at all (0)  🔾 Very rarely (1)  🔾 Rarely (2)  🔾 Sometimes (3)  🔾 Often (4)  🔾 Very often (5)  🔾 Almost always (6)  🔾 Not at all (0)  🔾 Very rarely (1)  🔾 Rarely (2)  🔾 Sometimes (3)  🔾 Often (4)  🔾 Very often (5)  🔾 Almost always (6)  🔾 Not at all (0)  🔾 Very rarely (1)  🔾 Rarely (2)  🔾 Sometimes (3)  🔾 Often (4)  🔾 Very often (5)  🔾 Almost always (6)  🔾 Not at all (0)  🔾 Very rarely (1)  🔾 Rarely (2)  🔾 Sometimes (3)  🔾 Often (4)  🔾 Very often (5)  🔾 Almost always (6)  🔾 Not at all (0)  🔾 Very rarely (1)  🔾 Rarely (2)  🔾 Sometimes (3)  🔾 Often (4)  🔾 Very often (5)  🔾 Almost always (6)  🔾 Not at all (0)  🔾 Very rarely (1)  🔾 Rarely (2)  🔾 Sometimes (3)  🔾 Often (4)  🔾 Very often (5)  🔾 Almost always (6)  🔾 Not at all (0)  🔾 Very rarely (1)  🔾 Rarely (2)  🔾 Sometimes (3)  🔾 Often (4)  🔾 Very often (5)  🔾 Almost always (6) |
| GAD-2 (Generalized Anxiety Disorder)  How often in the past 2 weeks have you been affected by the following problems?   a) Nervousness, anxiety, or tension b) Not being able to stop or control worrying | 🔾 Not at all  🔾 On some days  🔾 More than half of the days  🔾 Nearly every day  🔾 Not at all  🔾 On some days  🔾 More than half of the days  🔾 Nearly every day |
| PHQ-2  How often in the past 2 weeks have you been affected by the following problems?   a) Little interest or pleasure in doing things b) Feeling down, depressed, or hopeless | 🔾 Not at all  🔾 On some days  🔾 More than half of the days  🔾 Nearly every day  🔾 Not at all  🔾 On some days  🔾 More than half of the days  🔾 Nearly every day |
| Do you feel adequately supported overall with regard to your COPD? | 🔾 1 Strongly disagree  🔾 2  🔾 3  🔾 4  🔾 5 Strongly agree |
