# Supplementary material for: Needs among persons with advanced COPD and their informal caregivers: a multi-perspective qualitative study focusing on the potential role of palliative care
Source: BMC Pulm Med. 2026 Oct 19;26:484. doi: 10.1186/s12890-026-04828-5 (PMC13681904; doi:10.1186/s12890-026-04828-5)
Supplement: Supplementary file 6 [file 12890_2026_4828_MOESM6_ESM.docx]

**“Care Needs in advanced COPD: The perspective of patients, informal, and formal caregivers”**

Interview guide for informal caregivers, version 07 (as of 15.02.2024)

**______________________________________________________________________________**

| **Thematic blocks** | **Interview questions** |
| --- | --- |
| **Opening question**  **Experience of the disease in daily life** | Your [relation to the person, e.g., father] has COPD. Please tell me: how do you experience daily life with them, especially regarding their illness?   - *How did the disease start? How has it developed over time?* - *What limitations do you observe in the patient’s daily life as a result?* - *What tasks have you taken on since [person] developed COPD? How has this changed over time?* - *Is there anyone else who supports [person with COPD] in daily life?*   - *If yes, who does what?* - *Is [person] in need of care?*   - *If yes, who provides this care?*     - *If you provide the care: what exactly do you do? How time-consuming is it?* - *How has your life changed as a result of [person] having COPD?* - *Do you personally experience limitations due to [person]’s COPD? For example, regarding your time, your own health, your social life, etc.?* - *What feelings does [person]’s COPD evoke in you?* |
| **Medical needs**  Communication/exchange  Evaluation  Information | [Person] receives medical care for COPD. Do you know which physicians are treating [person] for COPD?   - *Are you also a point of contact for these physicians?* - *How do you experience the communication between [person] and these physicians?* - *How do you experience the communication between yourself and these physicians?* - *How could this communication be improved?*   How do you evaluate the medical support [person] receives regarding COPD?   - *In your opinion, what additional medical support would [person with COPD] need to feel better?* - *Are there specific challenges or concerns on your side regarding the current treatment of [person with COPD]?* - *Do you feel that [person] is in good medical hands? Why?*   Do you feel sufficiently informed about how to support [person] in daily life regarding COPD?   - *What information is missing or could help you (e.g., medication administration, use of medical devices)?* - *Who could/should provide you with this information? How? When?*   Do you feel sufficiently informed about COPD in terms of disease progression?   - *What information is missing or could help you?* - *Who could/should provide you with this information? How?*   Are there any questions or topics regarding [person]’s COPD and its impact on their life that you would like to discuss with the physician or other healthcare professionals? |
| **Organisational / social needs**  Assessment for patient  Information  Assessment for themselves | You have told me how you experience [person]’s COPD in daily life. What (additional) support do you need in order to better support [person]?   - *Who could provide this (additional) support to you? In what form?*   Do you feel sufficiently informed about how you, as an informal caregiver, can receive support (e.g., applications for care allowances)?   - *If no: what information would you like to have?*   - *From whom? In what form?* - *If yes: what do you find helpful?*   - *Where did you get this information from?*   Caring for a seriously ill relative can be very challenging. How do you experience this?  What (additional) support do you need/wish for in order to feel better yourself?   - *What is missing?* - *Who could provide this (additional) support? How?* - *What/who currently helps you? What could be improved?*   *Do you feel sufficiently informed about how you personally can receive support as an informal caregiver (e.g., self-help groups)?*   - *If no: what information would you like to have?*   - *From whom?* - *If yes: what do you find helpful?*   - *Where did you get this information from?* |
| **Psychological / spiritual needs**  Managing feelings  [If limitations were mentioned]  Dealing with disease progression | You told me that [person]’s COPD evokes [feelings] in you. How do you cope with these feelings?   - *What helps you cope with these feelings?* - *What else would you need to better manage these feelings?* - *Who could support you?* - *How could this support look like?*   *You mentioned that [person]’s COPD limits you.*   - *What would you need to be less affected?* - *Who could help you with this?* - *How could this support look like?*   What would it mean for you if [person]’s health further deteriorated due to COPD?   - *Do you have worries or concerns regarding the progression of [person]’s disease?*   - *If yes, what are they?*   - *What helps you cope with [person]’s disease progression?*   - *What else would help you cope better?* - *What support would you like to better handle [person]’s COPD progression?* - *Do you think [person] needs support in dealing with disease progression?*   - *If yes, what kind of support?*   - *And from whom?* |
| **[If exacerbations were mentioned]** | You have already talked about your experience with exacerbations. What information would help you better deal with them?   - *Who could provide this information?* - *Is there anything that has helped you be better prepared for exacerbations?* - *What form of support would help you cope better with exacerbations (e.g., medical, psychological, organizational, other)?*   - *Who could provide this support?* |
| **Palliative care**  [For informal caregivers of patients recruited via general practitioners and pulmonologists] | What is your immediate thought when you hear the word “palliative care”?  Palliative care is a form of medical treatment aimed at relieving symptoms and improving the quality of life of patients, enabling a better life despite serious illness. This includes attempts to control symptoms such as pain, shortness of breath, anxiety, or psychological suffering. Do you have any experience with palliative care?   - *If yes, when and how did you learn about it?*   - *Is [person] currently receiving palliative care?*   - *How helpful do you consider palliative care?*   - *Do you feel sufficiently involved in [person]’s palliative care, e.g., regarding decision-making?*     - *If no: what would you wish for?* - *If no, what do you think palliative care could do for [person]?*   - *If yes, in what way exactly?*   - *If no, why not?* - *Would you like to be informed about palliative care?* - *Do you have concerns regarding palliative care?*   - *If yes, what are they?* - *From when do you think palliative care would be helpful for [person]?* |
| **Palliative care**  [For informal caregivers of patients recruited via palliative care physicians] | [Person] is currently receiving palliative care for COPD. When, how, and from whom did you learn about [person]’s palliative care?   - *How helpful do you think the palliative care is for [person]?*   - *In what way?* - *How did [person] come to receive palliative care?* - *Did you have any specific worries or concerns when [person] decided on palliative care?* - *How has [person]’s palliative care affected you?*   - *In what way were you relieved or burdened? How?* - *Do you feel sufficiently involved in [person]’s palliative care?*   Do you have any wishes regarding [person]’s palliative care? / Could anything be done differently or better? |
| **Other needs** | Looking back: what would you have wished for when you first learned about [person]’s COPD? (e.g., more information about the disease, contact points, more support offers for informal caregivers)  Are there any other needs on your part regarding COPD that we have not yet discussed? |

| Your gender? | 🔾 Male  🔾 Female  🔾 Diverse  🔾 [Does not know]  🔾 [No response] |
| --- | --- |
| In which year were you born? | __  🔾 [Does not know]  🔾 [No response] |
| Country of birth | 🔾 Germany (current boundaries)  🔾 Another country:_____________________  🔾 [Does not know]  🔾 [No response] |
| Country of birth of your parents (multiple answers possible) | 🔾 Germany (current boundaries)  🔾 Another country:_____________________  🔾 [Does not know]  🔾 [No response] |
| Marital status:  *“Married,” “Divorced,” and “Widowed” also apply to registered same-sex partnerships. “Separated” means legally separated, not just living apart.* | 🔾 Married / in a partnership  🔾 Married, separated from spouse/partner  🔾 Single  🔾 Divorced  🔾 Widowed  🔾 [Does not know]  🔾 [No response] |
| Does the person with COPD live in your household? | 🔾 Yes, permanently  🔾 Yes, but not permanently (e.g., only on weekends)  🔾 No  🔾 [Does not know]  🔾 [No response] |
| Are there other people in your household who depend on your care/support? *If yes, please indicate the number and the persons (e.g., “two children”)* | 🔾 Yes, _______________________________  🔾 No  🔾 [Does not know]  🔾 [No response] |
| Do you have a serious illness diagnosed by a physician? | 🔾 Yes, ___________________________________  🔾 No  🔾 [Does not know]  🔾 [No response] |
| Where do you live? | 🔾 In a large city (> 100,000 inhabitants)  🔾 In a small city (20,000 – 100,000 inhabitants)  🔾 In a rural area (< 20,000 inhabitants) |
| *Highest* educational degree | 🔾 Student, attending a full-time general education school  🔾 Left school without completing secondary education  🔾 Secondary school leaving certificate (Hauptschule)  🔾 Intermediate school leaving certificate (Realschule / Mittlere Reife)  🔾 Polytechnic secondary school of the former GDR (8th–10th grade)  🔾 Technical college entrance qualification (Fachhochschulreife)  🔾 General or subject-specific higher education entrance qualification (Abitur / Gymnasium / EOS, including EOS with vocational training)  🔾 Other school degree  🔾 [Does not know]  🔾 [No response] |
| Are you employed? | 🔾 Yes  🔾 Employed full-time  🔾 Employed part-time  🔾 No  🔾 [Does not know]  🔾 [No response] |
| GAD-2 (Generalized Anxiety Disorder)  How often in the past 2 weeks have you been affected by the following problems?   a) Nervousness, anxiety, or tension b) Not being able to stop or control worrying | 🔾 Not at all  🔾 On some days  🔾 More than half of the days  🔾 Nearly every day  🔾 Not at all  🔾 On some days  🔾 More than half of the days  🔾 Nearly every day |
| PHQ-2  How often in the past 2 weeks have you been affected by the following problems?   a) Little interest or pleasure in doing things b) Feeling down, depressed, or hopeless | 🔾 Not at all  🔾 On some days  🔾 More than half of the days  🔾 Nearly every day  🔾 Not at all  🔾 On some days  🔾 More than half of the days  🔾 Nearly every day |
| Do you feel sufficiently supported in your role as a caregiver of a person with advanced COPD? | 🔾 1 Strongly disagree  🔾 2  🔾 3  🔾 4  🔾 5 Strongly agree |
