# Supplementary material for: Needs among persons with advanced COPD and their informal caregivers: a multi-perspective qualitative study focusing on the potential role of palliative care
Source: BMC Pulm Med. 2026 Oct 19;26:484. doi: 10.1186/s12890-026-04828-5 (PMC13681904; doi:10.1186/s12890-026-04828-5)
Supplement: Supplementary file 7 [file 12890_2026_4828_MOESM7_ESM.docx]

**“Care Needs in advanced COPD: The perspective of patients, informal, and formal caregivers”**

Interview Guide for General Practitioners and Pulmonologists, Version 07 (as of 15.02.2024)

**______________________________________________________________________________**

| **Thematic blocks** | **Interview questions** |
| --- | --- |
| **Opening question**  COPD Patients | You care for patients with COPD. What experience do you have in caring for patients with advanced COPD?   - Is it possible to characterize these patients in any way?   - *If so, how?* |
| **Health Care for Patients with advanced COPD**  Needs  Challenges/barriers  Facilitating factors  Information | In your own words, can you explain what “advanced COPD” means to you?  How do you determine this stage of disease in patients with COPD?  From your perspective, what specific needs do patients with advanced COPD have?   - *Who can or should meet these needs? How and at what point in time?*   How do you determine this stage of the disease in patients with COPD?  From your perspective, what specific needs do patients with advanced COPD have?   - *Who can or should meet these needs, and how and at what point in time?*   What challenges or barriers have you encountered in the care of patients with advanced COPD?   - *How could these barriers potentially be overcome?*   Which factors have you perceived as facilitating appropriate care for patients with advanced COPD?  What information do patients, as well as their informal caregivers, receive about prognosis and treatment options?   - *When do patients and/or informal caregivers receive this information?* - *In your opinion, how could communication about prognosis and treatment options for this target group be improved?* |
| **Palliative care**  Timing  Experience  Need for palliative care in advanced COPD  Specialized Outpatient Palliative Care (SOPC)  Information and education  Barriers | From your perspective, at what point would a palliative care concept be important for these patients?   - *Who should provide this care and inform patients about it?* - *How should patients be informed about it?* - *What should such a palliative care concept include?*   In your own words, can you describe what you understand by palliative care?   - *What goals does palliative care have from your perspective?* - *Do you yourself have experience with palliative care?*   - *If yes, also with patients with COPD?*   In your opinion, what is the need for palliative care among patients with advanced COPD?   - *Who addresses this need? How and when?*   How often have you prescribed SOPC for patients with COPD, and what goals were you pursuing?  Do you inform patients with advanced COPD about the possibility of palliative care?   - *Do you provide education to patients with advanced COPD about palliative care?*   - *If yes, how?*   - *If no, why not?*   - *How does this education differ from, for example, that provided to people with oncological diseases?*   - *At what point do you inform patients with COPD about palliative care?*   - *How much time do you need to provide education on palliative care to patients with advanced COPD? Do you have this time available?* - *What information would patients and their informal caregivers need in order to decide whether and from when palliative care would be helpful?*   - *Who could provide this information?*   What barriers do you see for patients with COPD in accessing palliative care?   - *Why do these barriers exist particularly for patients with COPD?* - *How could these barriers be reduced in order to improve access to palliative care for this target group?* |
| **Informal care**    Needs  Resources | In your opinion, what role do informal caregivers play in the care of patients with advanced COPD?  What type of support do informal caregivers of patients with advanced COPD need?   - How could informal caregivers be better supported? - What resources and information are available to you for informal caregivers? - Who, in your opinion, could serve as a contact person for informal caregivers? |
| **Additional needs of patients with advanced COPD** | From your perspective, what additional medical needs related to COPD—currently not covered by medical [and/or nursing] staff—exist for patients with advanced COPD?  Is there a need for further support for patients and their informal caregivers in connection with COPD (e.g., psychological or spiritual support, social counselling, financial assistance)?   - If yes, what could this look like? - Who should provide this support?   Are there any other forms of need that you observe among patients with advanced COPD that we have not yet discussed? |

| Your gender | 🔾 Male  🔾 Female  🔾 Divers  🔾 [Does not know]  🔾 [No response] |
| --- | --- |
| In which year were you born | ______  🔾 [No response] |
| In which role do you provide care for patients with advanced COPD? (multiple options possible) | 🔾 General practitioner  🔾 Pulmonologist  🔾 Other: _____________________________  🔾 [No response] |
| Are you also a palliative care physician? | 🔾 yes  🔾 no |
| How do you assess your own experience with COPD? | 🔾 1 – No experience at all  🔾 2  🔾 3  🔾 4  🔾 5 – Very experienced |
| How many years have you been practicing medicine? | ________ years |
| To what extent do you agree with this statement? | „Patients with end-stage COPD receive adequate palliative care.“  🔾 1 – Fully agree  🔾 2  🔾 3  🔾 4  🔾 5 – Fully disagree |
