# Supplementary material for: Needs among persons with advanced COPD and their informal caregivers: a multi-perspective qualitative study focusing on the potential role of palliative care
Source: BMC Pulm Med. 2026 Oct 19;26:484. doi: 10.1186/s12890-026-04828-5 (PMC13681904; doi:10.1186/s12890-026-04828-5)
Supplement: Supplementary file 8 [file 12890_2026_4828_MOESM8_ESM.docx]

**“Care Needs in advanced COPD: The perspective of patients, informal, and formal caregivers”**

Interview Guide for palliative care physicians, version 07 (as of 15.02.2024)

**______________________________________________________________________________**

| **Thematic blocks** | **Interview questions** |
| --- | --- |
| **Opening question**  COPD Patients | You care for patients with severe, incurable illnesses. What experience do you have with the palliative care of patients with advanced COPD?   - Is it possible to characterize these patients in any way?   - *If so, how?* |
| **Health care for patients with advanced COPD**  Needs  Challenges/barriers  Facilitating factors  Treatment | In your own words, can you explain what “advanced COPD” means to you?  From your perspective, what specific needs do patients with advanced COPD have?   - *Who can or should meet these needs? How and at what point in time?*   What challenges or barriers have you encountered in the care of patients with advanced COPD?   - *How could these barriers potentially be overcome?*   Which factors have you perceived as facilitating appropriate care for patients with advanced COPD?  What do you primarily focus on when treating patients with advanced COPD?   - *Do these patients receive any other specialized treatments tailored to their needs?* |
| **Palliative care**  Scope  Access  Timing  Need for patients with advanced COPD  Information  Barriers  Communication | Could you describe what palliative care encompasses in your daily work?   - *What does palliative care for patients with advanced COPD look like? What does it include?* - *How do patients with advanced COPD benefit from palliative care, or in which areas is palliative care particularly valuable?*   How did these patients come to be under your care?  Do you believe that palliative care is offered to patients with advanced COPD in a timely manner?   - If not, why has this not been possible so far? - When should palliative care be offered to patients with advanced COPD, and by whom?   - How would you determine such a timing? - How could patients be integrated into palliative care earlier?   In your opinion, what is the need for palliative care for patients with advanced COPD?   - *Which aspects of the intended palliative care for patients with advanced COPD do you consider adequate?* - *Which aspects are not adequate, or what is currently lacking?*   In your opinion, what information is necessary for patients with advanced COPD and their informal caregivers regarding palliative care?   - Do patients with advanced COPD have a particular need for information in order to decide if and when palliative care would be helpful?   - *If so, what kind of information?* - Do you provide this information? - From whom and where should patients with severe COPD receive information about palliative care for the first time?   What barriers do you see for patients with advanced COPD in accessing palliative care?   - *Why do these barriers exist specifically for COPD patients?* - *How could these barriers be overcome to improve access to palliative care for this target group?*   How do you communicate with patients with advanced COPD and their informal caregivers to understand their needs and wishes?   - *How do you inform patients and informal caregivers about palliative care interventions?* - *What informational materials are available to you for this target group?*   - *If you do not have any informational materials: do you see a need for such materials?*   - *If so, what kind of materials would be useful?* - *In your opinion, how could communication about palliative care for this target group be improved?* |
| **Informal care**  Needs  Resources | In your opinion, what role do informal caregivers play in the care of patients with advanced COPD?  What type of support or information do informal caregivers of patients with advanced COPD need?   - *How could informal caregivers be better supported?* - *What resources and information are available to you for informal caregivers?* |
| **Additional needs of patients with advanced COPD** | From your perspective, what additional (palliative) medical needs related to COPD—currently not covered by medical [and/or nursing] staff—exist for patients with advanced COPD?  Is there a need for further support for patients and their informal caregivers in connection with COPD (e.g., psychological or spiritual support, social counselling, financial assistance)?   - If yes, what could this look like? - Who should provide this support?   Are there any other forms of need that you observe among patients with advanced COPD that we have not yet discussed? |

| Your gender? | 🔾 Male  🔾 Female  🔾 Divers  🔾 [Does not know]  🔾 [No response] |
| --- | --- |
| In which year were you born? | ______  🔾 [No response] |
| How do you assess your own experience with COPD? | 🔾 1 – No experience at all  🔾 2  🔾 3  🔾 4  🔾 5 – Very experienced |
| How many years have you been practicing medicine? | ________ years |
| How many years have you been working in palliative care? | ________ Jahre |
| To what extent do you agree with this statement? | „Patients with end-stage COPD receive adequate palliative care.“  🔾 1 – Fully agree  🔾 2  🔾 3  🔾 4  🔾 5 – Fully disagree |
